# Oxidative Addition of Aryl Bromides at Palladium(I) to form Palladium(III) Complexes

**DOI:** 10.1002/anie.202514101

**Published:** 2025-08-26

**Authors:** Bailey S. Bouley, Dae Young Bae, Wen Zhou, Leonel Griego, Liviu M. Mirica

**Affiliations:** ^1^ Department of Chemistry University of Illinois at Urbana‐Champaign Urbana Illinois 61801 USA; ^2^ Department of Chemistry Washington University in St. Louis St. Louis Missouri 63130 USA

**Keywords:** Cryo stopped‐flow UV–vis spectroscopy, Mechanistic studies, Oxidative addition, Palladium, Paramagnetic Pd complexes

## Abstract

Herein, we report the first systematic study of the oxidative addition of aryl bromides to a Pd^I^ center to generate organometallic Pd^III^ complexes. These isolable Pd^III^ complexes stabilized by tetradentate macrocyclic pyridinophane ligands exhibit distinct UV–vis and EPR spectroscopic signatures that allowed for the monitoring of their generation in situ. These ligand scaffolds were sterically and electronically tuned using a modular synthetic approach to probe the kinetic properties and activation parameters of the oxidative addition reaction, and a combination of UV–vis and cryo stopped‐flow spectroscopic studies reveal a rapid oxidative addition step occurring at a Pd^I^ center. In addition, these results are in strong agreement with our recent reactivity studies, which demonstrated that mononuclear Pd^I^ systems are competent catalysts in Kumada cross‐coupling reactions, and thus set the stage for an improved understanding of potential catalytic applications for odd‐electron Pd systems.

## Graphical abstract

This work reveals a detailed mechanistic study of the oxidative addition of aryl bromides to a Pd(I) center to generate an isolable Pd(III) species. UV–vis and cryo stopped‐flow kinetic studies, including Eyring and Hammett analyses, reveal a rapid oxidative addition step occurring at a Pd(I) center, and a zero‐order dependence on the substrate.

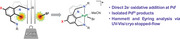

## Introduction

Oxidative addition (OA) is a key step in the transition metal‐mediated conversion of organic substrates into value‐added materials. This process generates an organometallic intermediate, which may undergo subsequent transmetalation and reductive elimination to form desirable C─C or C‐heteroatom bonds. Particularly, oxidative addition of organic electrophiles (RX, typically aryl halides) to Pd^0^ is of great synthetic importance and has been extensively studied in many cross‐coupling reactions such as Heck, Suzuki, Negishi, Stille, Sonogashira, Kumada, and Buchwald–Hartwig reactions, which are commonly catalyzed in Pd^0^/Pd^II^ cycles.^[^ ^1^, ^2^, ^3^, ^4^, ^5^, ^6^, ^7^, ^8^, ^9^, ^10^ ^]^ Boosted by the 2010 Nobel Prize to Pd‐catalyzed cross coupling reactions, catalytic transformations facilitated through Pd^II^/Pd^IV^ cycles have been developed as alternative pathways to the universal Pd^0^/Pd^II^ cycles for the past decade.^[^ ^11^, ^12^, ^13^, ^14^ ^]^ In all of these cycles, oxidative addition is driven by the electron‐rich state (either Pd^0^ or Pd^II^), after which transmetalation generates the unstable, electron‐rich bis‐organometallic intermediate, driving reductive elimination and subsequent bond formation.^[^ ^15^ ^]^ Recently, the significantly less common odd oxidation states of Pd have gained traction in the catalysis literature, especially in the realm of bond‐forming reactions and photochemistry.^[^ ^16^, ^17^, ^18^, ^19^ ^]^ While the chemistry of the dimeric forms of Pd^I^ and Pd^III^ have been well‐characterized,^[^ ^20^, ^21^, ^22^, ^23^, ^24^, ^25^, ^26^ ^]^ the chemistry of the mononuclear variants remains relatively elusive.

**Figure 1:**
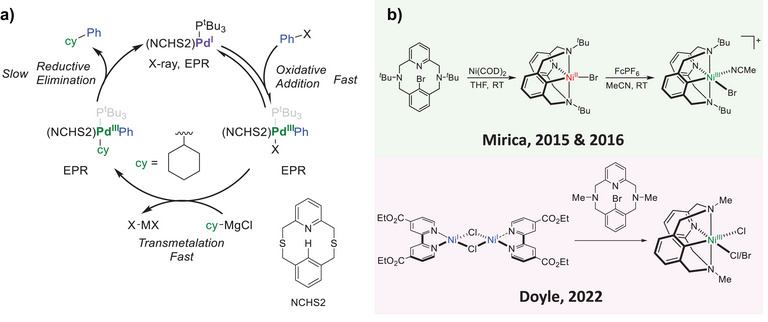
a) Mechanism of Csp^2^‐Csp^3^ Kumada cross coupling reactions mediated by Pd^I^ compounds supported by thioether‐based pyridinophane macrocyclic ligands. b) The use of ^R^N3CBr ligands for the stabilization of high‐valent organometallic Ni^III^ complexes.

Starting in 2010, our group showed that the Pd^III^ oxidation state may be stabilized through the use of polydentate macrocyclic ligands, which allowed for the isolation of the first mononuclear organometallic Pd^III^ complexes and their isolated reactivity.^[^ ^27^, ^28^, ^29^, ^30^, ^31^, ^32^, ^33^, ^34^, ^35^ ^]^ Mononuclear Pd^I^ species, on the other hand, are a much more recent development, and our understanding of their isolated reactivity is limited. To date, only 8 mononuclear Pd^I^ complexes have been crystallographically characterized. The first two, reported independently by the Chaplin and Ozerov groups in 2016, were the homoleptic Pd^I^(P^t^Bu_3_)_2_^+^ and its solvated analog Pd^I^(P^t^Bu_3_)_2_(MeCN)^+^.^[^ ^36^, ^37^ ^]^ The NHC analog Pd^I^(IPr)_2_^+^ was synthesized later in 2023 by de Jesús and coworkers, and this complex showed distinct and reversible oxygen reactivity.^[^ ^38^ ^]^ The original Pd^I^ report was followed up by Deng and coworkers in 2021 who isolate the first heteroleptic Pd^I^ complex supported by a bisphosphine ligand and a redox‐active aryl amide.^[^ ^39^ ^]^ Recently, Gade and coworkers employed a PNP carbazole‐based pincer ligand to isolate a T‐shaped Pd^I^ complex capable of reactivity with CO and disulfides to generate thioester moieties.^[^ ^40^ ^]^ Finally, a recent preprint, again by Deng, synthesized a two‐coordinate, thermally stable NHC‐Pd^I^‐aryl complex which showed interesting radical‐based Csp^2^‐Csp^3^ cross coupling reactivity, albeit stoichiometrically.^[^ ^41^ ^]^ One of the key missing pieces across these reports was a lack of catalytic reactivity. While a cross‐coupling reaction between aryl trifluoroborates and antimony‐based nucleophiles mediated by Pd^I^(P^t^Bu_3_)_2_^+^ was reported in 2018 by Chaplin and Hooper, this reaction necessitated specialized nucleophiles and stoichiometric oxidants to operate via a, likely, bimetallic Pd^0^/Pd^I^/Pd^II^ catalytic cycle.^[^ ^42^ ^]^ In 2022, our group reported the isolation of two new mononuclear Pd^I^ complexes supported by thioether‐based pyridinophane macrocycles, one of which was able to perform Csp^2^‐Csp^3^ cross coupling reactions rapidly under mild conditions (Figure 1a).^[^ ^43^ ^]^ Interestingly, this reaction appears to operate via a mononuclear Pd^I^/Pd^III^ catalytic cycle, akin to that typically observed in Ni‐mediated Csp^3^‐Csp^x^ couplings. While we gave tangible evidence for the direct oxidative addition process from Pd^I^ to Pd^III^ in the presence of aryl halides by EPR spectroscopy, a more systematic study was warranted to confirm this observation.

The inherent instability of catalytically‐relevant Pd^III^ complexes, generated post‐oxidative addition into Pd^I^ with exogeneous aryl halides makes their study difficult. However, we envisioned that using a ligand scaffold containing an aryl C─X bond that stabilizes high‐valent states may allow for direct analysis of the reaction. One potential avenue utilizes the ^R^N3CBr class of ligands (^R^N3CBr = 5^2^‐bromo‐3,7‐dialkyl‐3,7‐diaza‐1(2,6)‐pyridina‐5(1,3)‐benzenacyclooctaphane). Our group has previously used such ligands to isolate Ni^III^ complexes, prepared via oxidative addition into the zero‐valent precursor Ni^0^(COD)_2_ followed by one‐electron oxidation with a chemical oxidant.^[^ ^44^, ^45^, ^46^ ^]^ These complexes were mostly used to study high‐valent reductive elimination reactivity in C‐X bond forming reactions starting from the Ni^III^ state, however, the oxidative addition process at Ni^0^ or Ni^I^ was not analyzed in great detail (Figure 1b). The stability of ^R^N3C‐Pd complexes has also been studied previously, however, their preparation has typically used an alternative route involving the ^R^N3CH‐type ligands, where direct C─H bond activation generates the organometallic Pd^II^ complex by non‐oxidative routes.^[^ ^32^ ^]^ We envisioned that Pd^III^ complexes could be directly generated in situ via the oxidative addition of a Pd^I^ starting source into a ^R^N3CBr‐type macrocycle, which would skip the need for a step‐wise preparation and allow us to probe in detail the oxidative addition process at a Pd^I^ center. In addition, in 2022 Doyle et al. have used the ^Me^N3CBr ligand to probe the in situ formation of Ni^I^ species that can undergo oxidative addition to make an isolable Ni^III^ complex.^[^ ^47^ ^]^ In any case, this approach has never been applied to Pd^I^ compounds. Herein, we demonstrate that electronically‐ and sterically‐modulated *p*‐X^R^N3CBr ligands can be used to probe the oxidative addition process at Pd^I^, to generate isolable Pd^III^ organometallic compounds. Improving our understanding of the reactivity from this oxidation state may be critical in the development of alternative Pd‐mediated coupling reactions or accessing new, more challenging substrates.

## Results and Discussion

### Synthesis and Characterization of Complexes

**Figure 2:**
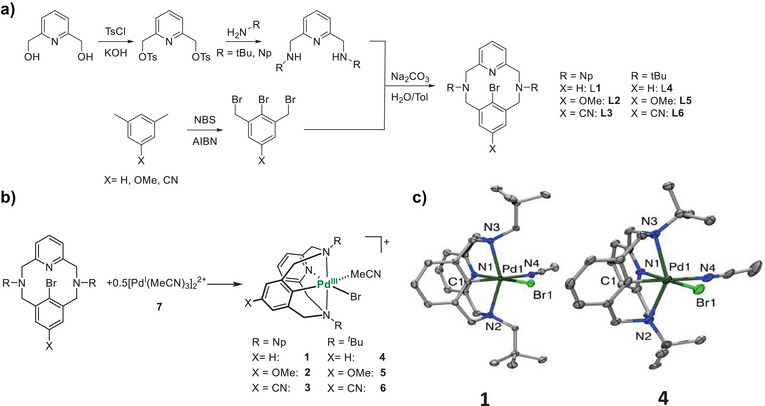
a) The generalized synthetic scheme for the preparation of ligands **L1**‐**L6**. b) The synthesis of isolated Pd^III^ compounds (**1**–**6**) supported by ligands **L1**‐**L6**. c) ORTEPs of complexes **1** and **4** shown at 50% probability. Selected bond distances (Å): **1**, Pd1‐C1, 1.951(2), Pd1‐N1, 2.046(1), Pd1‐N2, 2.343(1), Pd1‐N3, 2.338(1), Pd1‐N4, 2.193(1), Pd1‐Br1, 2.475(1), **4**, Pd1‐C1, 1.950(5), Pd1‐N1, 2.064(4), Pd1‐N2, 2.376(4), Pd1‐N3, 2.416(4), Pd1‐N4, 2.172(5), Pd1‐Br1, and 2.484(1).

The synthesis of the six ligand frameworks and the preparation of the Pd^III^ complexes used in this study are shown in Figure 2 (See Supporting Information for full synthetic details). The ligands are divided into two steric classes depending on the tertiary amine substituent: neopentyl (Np, **L1**‐**L3**) and *tert*‐butyl (^t^Bu, **L4**‐**L6**), with neopentyl representing a less sterically demanding fragment and *tert*‐butyl representing a more sterically demanding fragment. The steric profiles of these types of complexes have been studied previously.^[^ ^48^ ^]^ These two classes are further divided by their electronic profile, from the electron‐donating *p*‐OMe, to the neutral *p*‐H, and then the electron‐withdrawing *p*‐CN. By sampling electronic parameters across the σ_p_ range, we can establish the electronic structure of the oxidative addition process, as described further below.

**Figure 3:**
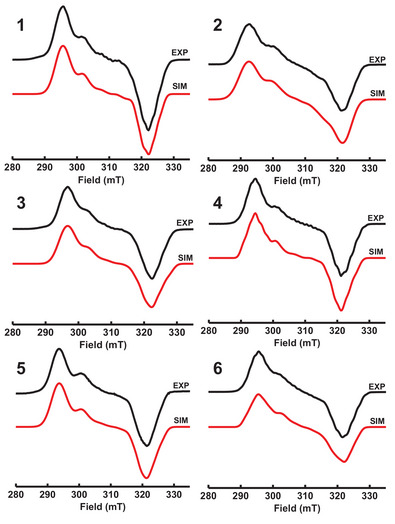
Experimental (PrCN glass, 77 K) and simulated EPR spectra of **1**–**6**. The following parameters were used for simulations: **1**, *g*_x_ = 2.196, *g*_y_ = 2.088 (A_N_ = 15.0 G, A_Br_ = 47.0 G), *g*_z_ = 2.018 (A_2N_ = 18.0 G), **2**, *g*_x_ = 2.210, *g*_y_ = 2.092 (A_N_ = 16.0 G, A_Br_ = 49.0 G), *g*_z_ = 2.022 (A_2N_ = 22.0 G), **3**, *g*_x_ = 2.188 (A_2N_ = 13.0 G), *g*_y_ = 2.081 (A_N_ = 15.0 G, A_Br_ = 47.0 G), *g*_z_ = 2.017 (A_2N_ = 25.0 G), **4**, *g*_x_ = 2.204 (A_2N_ = 18.0 G), *g*_y_ = 2.092 (A_N_ = 16.0 G, A_Br_ = 50.0 G), *g*_z_ = 2.024 (A_2N_ = 22.0 G), **5**, *g*_x_ = 2.221 (A_2N_ = 13.0 G), *g*_y_ = 2.110 (A_N_ = 15.0 G, A_Br_ = 41.0 G), *g*_z_ = 2.021 (A_2N_ = 15.0 G), **6**, *g*_x_ = 2.197 (A_2N_ = 19.0 G), *g*_y_ = 2.095 (A_N_ = 8.0 G, A_Br_ = 39.0 G), and *g*_z_ = 2.017 (A_2N_ = 19.0 G).

When considering the Pd^I^ source to use for this study, several key considerations needed to be taken: 1) The starting material needed to be stable enough to prepare and isolate, but reactive enough to generate stable Pd^III^ compounds after oxidative addition with **L1**‐**L6**, 2) The starting material should avoid strongly associating ligands to prevent obfuscation of the final product spectroscopically. As such, the chosen Pd^I^ source was the acetonitrile‐coordinated dinuclear Pd^I^ complex [Pd^I^(MeCN)_3_]_2_(BF_4_)_2_ (**7**), which could easily be prepared via comproportionation of Pd^0^_2_(dba)_3_ with [Pd^II^(MeCN)_4_](BF_4_)_2_.^[^ ^49^ ^]^ Although this species is dimeric, it benefits from its isolability and stability, as well as a lack of strongly coordinated ligands among the sparsity of available Pd^I^ compounds. We envisioned that this precursor should be able to readily react with our ligands to generate mononuclear Pd^III^ complexes in a 2:1 ligand:dimer ratio. Gratifyingly, the reaction of **7** with ligands **L1**‐**L6** resulted in the generation of Pd^III^ complexes **1**‐**6** that could be straightforwardly characterized by EPR and UV–vis spectroscopy (Figures 3 and S25–S30). The observed EPR signals for the reactions with **L1** and **L4** were identical to those obtained from independently synthesized compounds **1** and **4**.^[^ ^50^ ^]^ The strong similarity in the electronic structure across compounds **1**–**6** by EPR indicates that the coordination environment around the Pd^III^ center is conserved across all these complexes (See Figure 2). The analogous structures of these compounds are also consistent with the UV–vis absorption features, where complexes bearing the neopentyl N‐substituents exhibit a strong absorption feature around 630 nm, and complexes bearing the *tert*‐butyl N‐substituents exhibit a strong absorption feature around 698 nm. As expected, varying the steric profile of the tertiary amine results in the most drastic spectral changes, as manipulating the energy level of the d_z_^2^‐derived HOMO from this axial interaction would result in large changes in these features.

### Kinetics of Oxidative Addition

**Figure 4:**
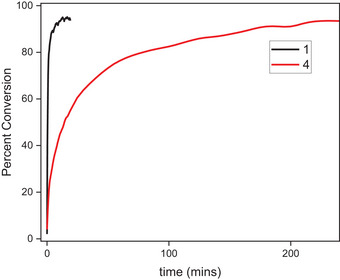
Representative kinetic traces for the reaction between **L1** or **L4** and **7** (2:1 = **L**:**7** ratio) at room temperature in a 1:1 mixture of MeCN:THF for complex **1** and **4** respectively.

Tracking the oxidative addition reaction of **7** into each of the ligands **L1**‐**L6** by UV–vis in a 50:50 mixture of MeCN:THF at room temperature allowed us to gain key insight into the rate of the oxidative addition process. In general, ligands bearing neopentylamine groups reacted with **7** in a 2:1 (**L**:**7**) ratio to form 2 equiv. of the relevant Pd^III^ complexes in significantly shorter reaction times as opposed to those bearing *tert*‐butyl groups (Figure 4). We anticipate that this is due to the higher degree of steric encumbrance exhibited by the *tert*‐butyl groups with respect to the Pd center. In the case of a concerted OA step, it would be expected that the rate of oxidative addition should increase in the presence of electron‐withdrawing substituents such as *p*‐CN relative to electron‐donating substituents.^[^ ^51^, ^52^ ^]^ However, initial evidence suggested that no such correlation was present regardless of amine substituent. Furthermore, fitting of the data curves by tracking for the formation of the features at 632 nm or 698 nm for **L1**‐**L3** and **L4**‐**L6** respectively proved challenging, as first‐order and second‐order reaction modeling resulted in poor experimental fits. This is possibly due to the dissociation of the dinuclear Pd^I^ precursor to generate 2 equiv. of mononuclear Pd^I^ being a step in the reaction, which could result in half‐order kinetics. To confirm this, initial rate experiments were performed by varying the ratio of **7** to each of the ligands (Figures 4 and S31–S36; Table S1). Across the **L1**‐**L5** ligands and excepting **L6** (which exhibited very slow reactivity), the oxidative addition reaction by initial rate determination appears to follow zero‐order dependence on ligand and a half‐order dependence on **7**, for an overall reaction order of 0.5 (rate = k[Pd]^0.5^[L]^0^). This is further supported by the somewhat similar rates for the **L1**‐**L3** and **L4**‐**L5** series of ligands, although these ligands exhibit different electronics. While the rate law does not explicitly depend on the ligand, differences in steric hindrance between ligands (e.g., neopentyl (**L1**) versus *tert*‐butyl (**L4**) in Figure 4) may influence the kinetic profiles and lead to slightly different rate constants. The existence of half‐order reactivity is often observed in cases where cleavage of dinuclear precursors is involved, and due to the overall reaction stoichiometry (1 equiv. **7** to 2 equiv. ligand), this is consistent with the proposed kinetic model.^[^ ^53^, ^54^, ^55^ ^]^

### Activation Parameters of Oxidative Addition

**Figure 5:**
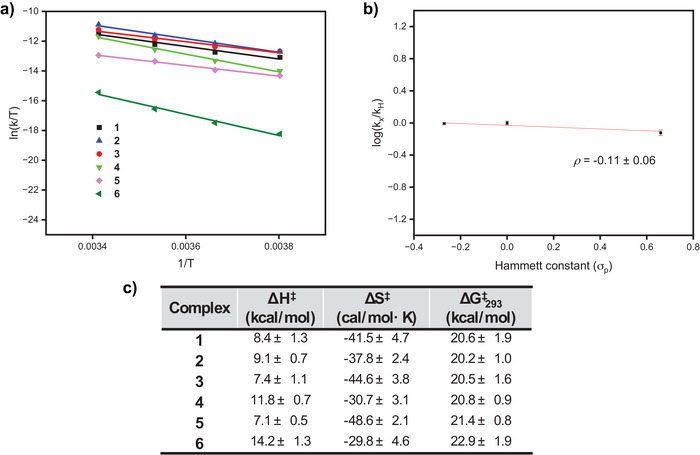
a) Eyring analysis for the reaction between **7** and ligands **L1**‐**L6** at 20 °C by cryo stopped‐flow UV–vis spectroscopy. b) Hammett analysis of the reaction between **7** and ligands **L1**‐**L3** determined by the initial rate method. Errors are propagated through 1 standard deviation from an average of 3 trials. c) Experimental Eyring parameters for the generation of **1**–**6** determined via cryo stopped‐flow UV–vis spectroscopy.

In order to gain further insight into the oxidative addition process, we turned to Eyring analysis to determine the kinetic parameters ΔG^‡^, ΔH^‡^, and ΔS^‡^, thus obtaining mechanistic insight into this reaction. Due to the rapid nature of these reactions, standard UV–vis was ill‐suited to give accurate enough data to obtain reliable Eyring parameters. Therefore, we decided to utilize cryo stopped flow UV–vis spectroscopy instead.^[^ ^56^ ^]^ Analyzing the reaction from −10 to 20 °C for ligands **L1**‐**L6** established trends consistent with the observed UV–vis reaction kinetics (Figure 5a). Ligands **L1**‐**L3** give very similar activation parameters, consistent with having similar reaction times and reaction orders.

In general, ligands **L1**‐**L5** give a distinct clustering, while **L6**, the slowest ligand to react with **7** to give the corresponding **6**, appeared to be an outlier. Ligands **L1**‐**L5** all demonstrate similar Δ*H* ^‡^ values ranging from 7.1–11.8 kcal mol^−1^ as well as large negative Δ*S* ^‡^ values ranging from −30.7 to −48.6 cal mol·K^−1^, with the neopentyl ligands exhibiting lower Δ*G* ^‡^_293_ values compared to those with *tert*‐butyl ligands (20.2–20.6 kcal mol^−1^ versus 20.8–22.9 kcal, Figure 5c). This ∼ 1 kcal mol^−1^ difference in Δ*G* ^‡^_293_ between the ligand groups is consistent with the observed difference reaction times (neopentyl ligands reacting within 5 min versus *tert*‐butyl ligands requiring reaction times on the hour time scale). The discrepancy between a largely positive Δ*S* ^‡^ value and an apparent 0.5 reaction order is discussed later in greater detail.

While the more negative Δ*S* ^‡^ values for ligands **L1**‐**L5** indicate a bimolecular rate determining step, the slightly less negative Δ*S* ^‡^ value for **L6** is borderline for such a process. A similar Δ*S* ^‡^ value is observed for reaction of **7** with **L4** but is less pronounced, likely owing to the change in Pd concentration dependence.

Hammett analysis was also performed on these reactions at 20 °C using the initial rates from the stopped‐flow UV–vis data to obtain a *rho* (*ρ*) value (Figure 5b). The neopentyl ligands show a very slightly negative *rho* value (*ρ* = −0.11 ± 0.06, Figure S39), indicating negligeable charge build‐up during the rate‐determining transition state. Encouragingly, the slightly negative *rho* value obtained for the neopentyl ligand greatly resembles the value obtained from the catalytic reactivity we previously reported for our isolated mononuclear Pd^I^ systems (*ρ* = −0.11),^[^ ^43^ ^]^ suggesting that the current mechanistic studies are relevant to Pd‐mediated catalytic processes.

**Figure 6:**
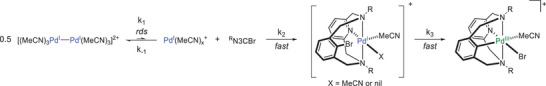
Proposed step‐wise mechanism for the oxidative addition reaction between **7** and ^R^N3CBr ligands.

Altogether, the kinetic data in conjunction with the reaction orders for ligands **L1**‐**L5** can be used to generate a mechanism for the oxidative addition process (Figure 6). We propose that the reaction proceeds through three major steps: i) the formation of a relatively low concentration of solvated Pd^I^ monomer from reversible cleavage of **7**, ii) complexation of this monomer with the ^R^N3CBr ligand, and iii) oxidative addition of C─Br bond to generate the final Pd^III^ complex. Among these, the rate determining step is the initial cleavage of dimer **7** to form the Pd^I^ monomer. Consistent with the rate law (rate = k[Pd]^0.5^[L]^0^, Table S1), only the dimer is involved in the RDS (half‐order dependence), with no apparent contribution from the ligand or other components.

In the Eyring plot (Figure 5c), the activation parameters, Δ*H* ^‡^, Δ*S* ^‡^, and Δ*G* ^‡^ are similar for all ligands, indicating that the transition state (TS) is independent of the ligand. The negative Δ*S* ^‡^ value results from solvent molecules coordinating to the Pd^I^ monomer formed upon Pd^I^─Pd^I^ bond cleavage, reducing entropy. DFT calculations further confirm that dimer dissociation is thermodynamically uphill (Δ*G* = 8.6–27.5 kcal mol^−1^), regardless of the number of solvent molecules involved in stabilizing the monomer (Figure S46), in agreement with experimental observations.

The Hammett plot supports the conclusion that the rate‐determining step is not influenced by the electronic properties of the pyridinophane ligands, as evidenced by the near‐zero *ρ* value. Additional competitive reactions with two electronically distinct ligands (**L2** and **L3**) and **7** were conducted (Tables S3 and S4; Figures S40–S43). After 10 seconds of the reaction, 7.4% of **L3** and 9.8% of **L2** were consumed. The consumption ratio (**L3**/**L2 **= 0.75) is consistent with that observed in initial rate measurements (0.77, Table S1). The measured *ρ* of −0.13 from this competitive reaction agrees with values derived from independent reactions (−0.11), indicating that electronic effects of the ligands have a minimal impact on the overall rate. Furthermore, the half‐order dependence on dimer **7** was confirmed: when 0.25 equiv. **7** was used, the initial rate decreased by half compared to the standard conditions (**7**:**L3**:**L2 **= 0.25:1:1, Table S4). These findings further support the conclusion that the pyridinophane ligand is not involved in the RDS to an appreciable extent, and thus the process follows zero‐order kinetics in ligand. The steps following the RDS proceed rapidly, involving the irreversible ligation of Pd^I^ monomer with ^R^N3CBr and subsequent fast oxidative addition to form a Pd^III^ complex. This mechanistic scenario is further supported by our study of heteroleptic Pd^I^ complexes with related pyridinophane ligands that undergo rapid oxidative addition to form Pd^III^ intermediates in a proposed Pd^I/III^ catalytic cycle.^[^ ^43^ ^]^

In summary, the proposed mechanism features a reversible cleavage of dimer **7** to generate a low concentration of solvated mononuclear Pd^I^ species, followed by ligand coordination and fast C‐Br bond oxidative addition, leading to the final Pd^III^ product (Figure 6). Notably, oxidation of Pd^I^ to Pd^III^ occurs more quickly than analogous processes in Pd^II^ and Ni^II^ systems employing the same ligand framework.^[^ ^32^, ^44^, ^45^, ^46^, ^47^, ^48^ ^]^

## Conclusion

Herein, we report the first experimental evidence for direct two‐electron oxidative addition of aryl halides to Pd^I^ centers, yielding stable organometallic Pd^III^ complexes supported by tetradentate macrocyclic pyridinophane ligands. Steric and electronic effects were elucidated through Hammett and Eyring analyses, using ligands bearing bulky *tert*‐butyl or less bulky neopentyl moieties, in combination with *p*‐OMe, *p*‐H, or *p*‐CN substituents on the aryl halide. The proposed mechanism involves: i) cleavage of a Pd^I^ dimer to form a Pd^I^ monomer, ii) complexation of this monomer with ^R^N3CBr, and iii) rapid C─Br oxidative addition. Notably, Pd^I^─Pd^I^ bond cleavage exerts a greater influence on the overall reaction timescale than the C─Br bond activation step. These findings provide valuable insight into the oxidative addition pathways of Pd^I^ complexes and should inform future efforts to develop Pd^I^‐based catalysis for accessing new and challenging substrates.

## Supporting Information

The data that support the findings of this study are available in the Supporting Information of this article. The authors have cited additional references within the Supporting Information.^[^ ^44^, ^45^, ^46^, ^49^, ^57^, ^58^, ^59^, ^60^, ^61^, ^62^, ^63^, ^64^, ^65^ ^]^

## Conflict of Interests

The authors declare no conflict of interest.

## Supporting information



Supporting Information

Supporting Information

## Data Availability

The data that support the findings of this study are available in the Supporting Information of this article.
