# Supplementary material for: Oxidative Addition of Aryl Bromides at Palladium(I) to form Palladium(III) Complexes
Source: Angew Chem Int Ed Engl. 2025 Aug 26;64(43):e202514101. doi: 10.1002/anie.202514101 (PMC12535386; doi:10.1002/anie.202514101)
Supplement: Supplementary file 1 — Supporting Information [file ANIE-64-e202514101-s001.pdf]

Supplementary Materials
for

Oxidative Addition of Aryl Bromides at Palladium(I) to Form Palladium(III) Complexes

Bailey S. Bouley,[a],# Dae Young Bae,[a],# Wen Zhou,[b] Leonel Griego,[a] and Liviu M. Mirica[a],*

[a] Department of Chemistry, University of Illinois at Urbana-Champaign, Urbana, Illinois, 61801.
[b] Department of Chemistry, Washington University, St. Louis, Missouri, 63130.

* Email: mirica@illinois.edu

# These authors contributed equally to this work.

Table of Contents

I. General Experimental Details S2
II. Synthesis of Ligands and Pd^{III} Complexes S3
III. Characterization Data S15
IV. Cryo Stopped-Flow UV-Vis Data S26
V. Solid State Structure Determinations S33
VI. Computational Details S42
VII. References S48

I. General Experimental Details

Reagents, Materials, and Physical Measurements

All manipulations were carried out under an inert atmosphere using a nitrogen-filled glovebox or standard Schlenk techniques unless otherwise noted. All reagents for which synthesis is not given were commercially available from Sigma, Acros, STREM, VWR or TCI and were used as received without further purification. All glassware was oven or flame-dried immediately prior to use. Tetrahydrofuran, MeCN, dichloromethane, toluene, *n*-pentane, and diethyl ether were degassed and dried by passage through a series of drying columns using a MBRAUN SPS Solvent System. All solvents were stored over 4 Å molecular sieves. Solvents were frequently tested using a standard solution of sodium benzophenone ketyl in tetrahydrofuran to confirm the absence of oxygen and moisture. Benzene-d_6, Acetone-d_6 and MeCN-d_3 were degassed and dried over 4 Å molecular sieves before use. All other reagents were dried and degassed thoroughly prior to use. **L1**, **L4**, and **L5** were synthesized following previously published procedures.[1-3] NMR spectra were recorded at ambient temperature, unless otherwise stated, on a Varian Mercury-300 400 MHz or a Bruker B600 NEO NMR spectrometer. ^{1}H and ^{13}C NMR chemical shifts were referenced to residual solvent and are reported in parts per million. Solution magnetic susceptibility measurements were obtained by the Evans method[4] in MeCN using coaxial NMR tubes at 293 K, and diamagnetic corrections were applied as previously described.[5-6] UV-vis spectra were recorded on a Varian Cary 50 UV-vis spectrophotometer using Cary WinUV software. EPR spectra were recorded on a JOEL JES-FA X-BAND (9.2 GHz) EPR spectrometer as a frozen *n*-PrCN glass at 77 K. ESI mass spectrometry (ESI-MS) studies were performed at the Washington University Mass Spectrometry Resource. Elemental microanalyses were performed by Intertek Pharmaceutical Services, Whitehouse, NJ. Cryo Stopped-Flow UV-Vis experiments were performed using a TgK Scientific CSF-61DX2 KinetAsyst Cryo Stopped-Flow System equipped with a 75W Xenon lamp. Reaction temperatures were maintained using a Hi-Tech KinetAsyst TC-61 Temperature Control Unit. Data was measured using a Hi-Tech Scientific KinetaScan Rapid Scanning CCD. Data obtained from these experiments was analyzed using the Kinetic Studio software suite.

II. Synthesis of Ligands and Pd^{III} Complexes

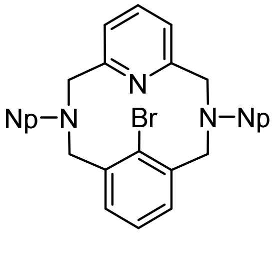

L1

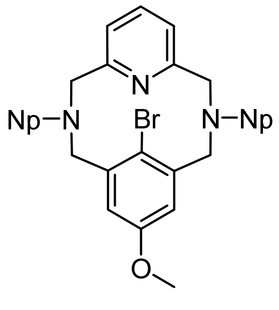

L2

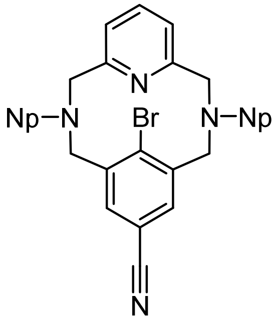

L3

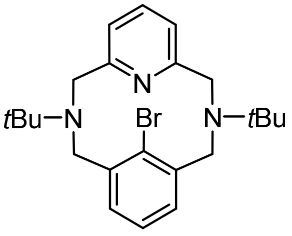

L4

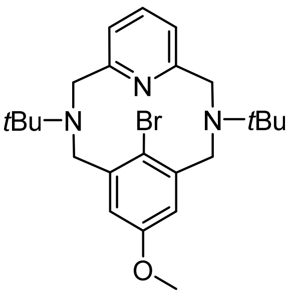

L5

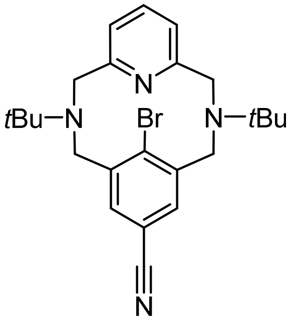

L6

Np = neopentyl and *t*Bu = *tert*-Butyl

Synthesis of 4-bromo-3,5-bis(bromomethyl)benzonitrile

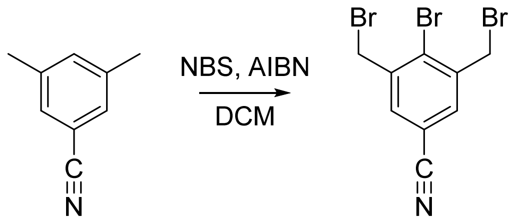

A 250 mL round bottom flask equipped with a magnetic stirring bar was charged with 4-bromo-3,5-dimethylbenzonitrile (0.20 g, 0.95 mmol, 1 equiv), NBS (0.68 g, 3.82 mmol, 4 equiv), and AIBN (0.08 g, 0.48 mmol, 0.5 equiv) under N_2 and 50 mL of degassed dry DCM was added. The reaction was stirred at room temperature for 24 hours under N_2. The solvent was removed and the solid was extracted with Et_2O (3×50 mL). The extracted reaction was purified using CombiFlash system (hexanes/ethyl acetate). 4-bromo-3,5-bis(bromomethyl)benzonitrile was isolated as a white powder. (yield: 87 mg, 25%).

^{1}H NMR (500 MHz, $CDCl_3$) δ 7.69 (s, 2H), 4.61 (s, 4H).

^{13}C NMR (126 MHz, $CDCl_3$) δ 140.43, 133.95, 131.88, 117.06, 112.59, 32.08, 31.06.

ESI-MS of 4-bromo-3,5-bis(bromomethyl)benzonitrile in MeCN: m/z 367.8097 (calcd for $C_9H_7Br_3N$, m/z 367.8103).

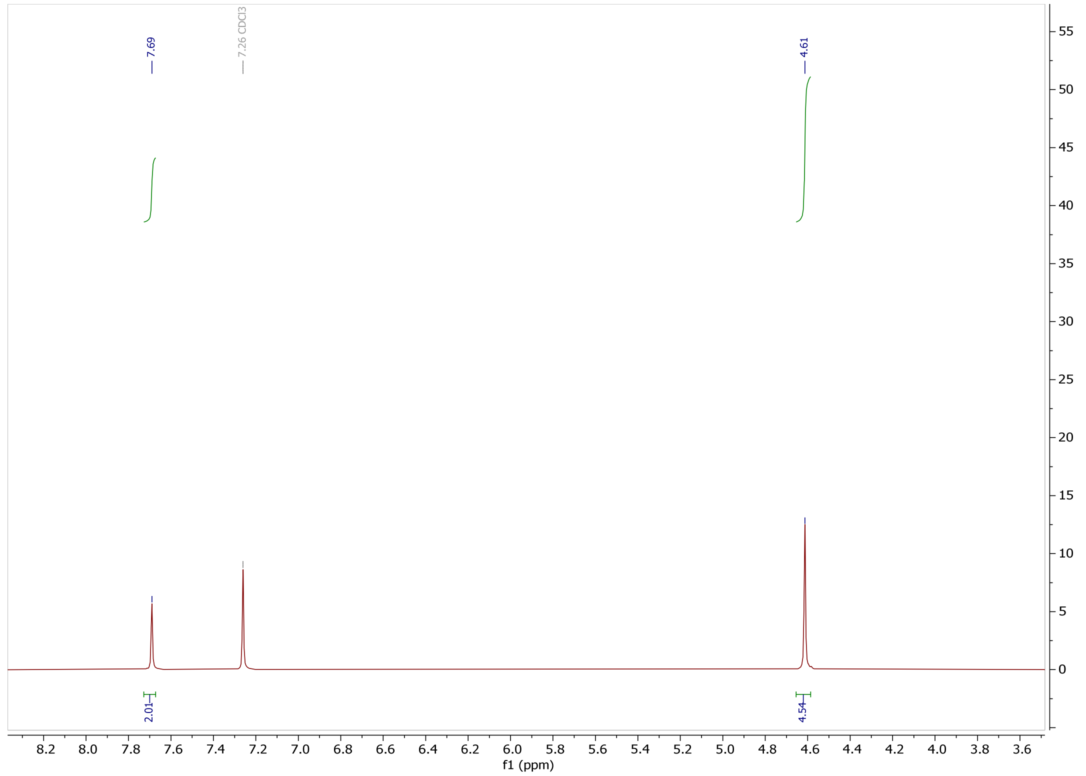

Figure S1. ^{1}H NMR of 4-bromo-3,5-bis(bromomethyl)benzonitrile in $CDCl_3$.

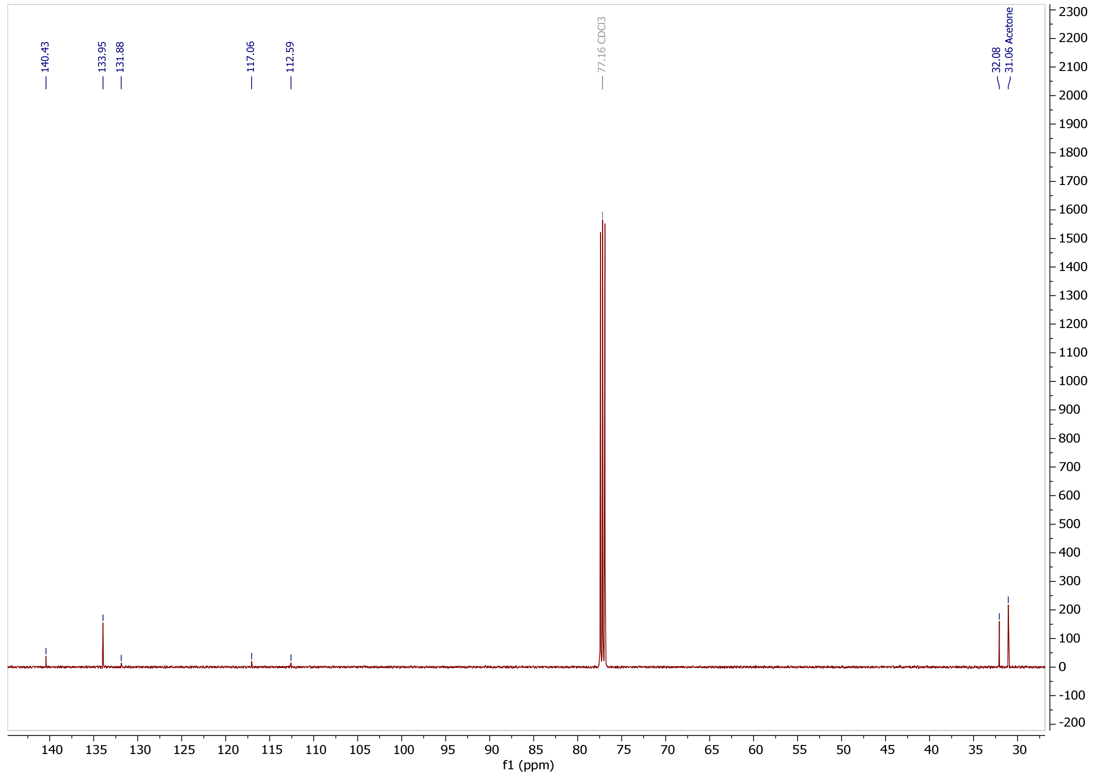

Figure S2. ^{13}C NMR of 4-bromo-3,5-bis(bromomethyl)benzonitrile in $CDCl_3$.

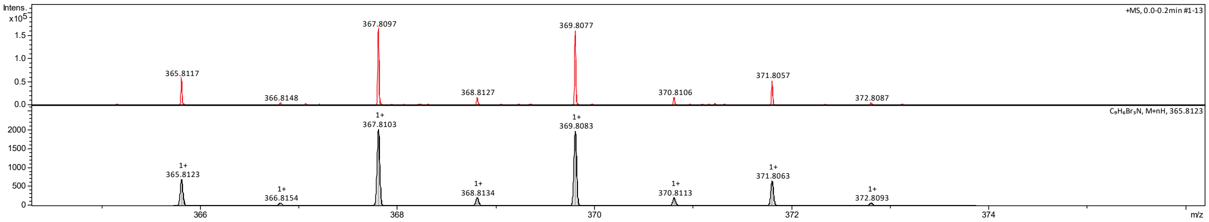

Figure S3. Experimental (top) and simulated (bottom) ESI-MS spectra of 4-bromo-3,5-bis(bromomethyl)benzonitrile in MeCN.

Synthesis of L2

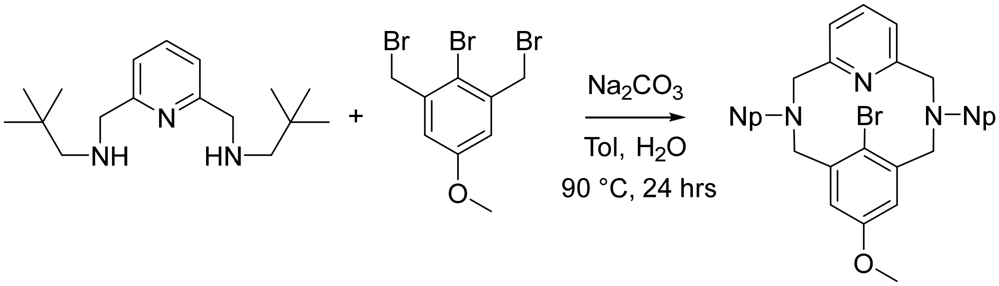

A 250 mL three-neck round bottom flask equipped with a reflux condenser, an addition funnel, and a magnetic stirring bar was charged with a solution of N,N'-(pyridine-2,6-diylbis(methylene))bis(2,2-dimethylpropan-1-amine) (0.48 g, 1.73 mmol, 1 equiv) in 100 mL of toluene and 60 mL of 40 % aqueous solution of Na_2CO_3. The stirred reaction mixture was pre-heated in an oil bath at 90 °C. A solution of 4-Bromo-3,5-bis(bromomethyl)anisole (0.65 g, 1.74 mmol, 1 equiv) in 30 mL toluene was added dropwise to the stirred reaction mixture at 90 °C over 2 hours. The resulting solution was heated at 90 °C under N_2 for an additional 24 hours. The reaction mixture was cooled down to RT, the organic layer was separated, and the solvent was removed in vacuo to generate a yellowish oil mixture. The yellowish yellow oil was extracted with pentane (3×5 mL). The solution was concentrated to ~5 mL and stored at -20 °C overnight. The solution was filtered to remove any precipitate. The solvent was removed to yield yellow oil. (yield: 800 mg, 94%).

^{1}H NMR (600 MHz, $CDCl_3$) δ 7.24 (t, J = 7.6 Hz, 1H), 6.97 (d, J = 7.6 Hz, 2H), 6.54 (s, 2H), 4.41 (d, J = 12.6 Hz, 2H), 3.91 (d, J = 13.0 Hz, 2H), 3.80 (d, J = 13.0 Hz, 2H), 3.68 (d, J = 12.8 Hz, 2H), 3.60 (s, 3H), 2.52 (q, J = 13.9 Hz, 4H), 1.07 (s, 18H).

^{13}C NMR (151 MHz, $CDCl_3$) δ 159.35, 157.07, 139.03, 134.61, 122.25, 117.76, 72.30, 66.79, 63.88, 55.11, 34.28, 28.24.

ESI-MS of **L2** in MeCN: m/z 488.2263 (calcd for [**L2**·H]$^+$, $C_{26}H_{39}BrON_3$, m/z 488.2271).

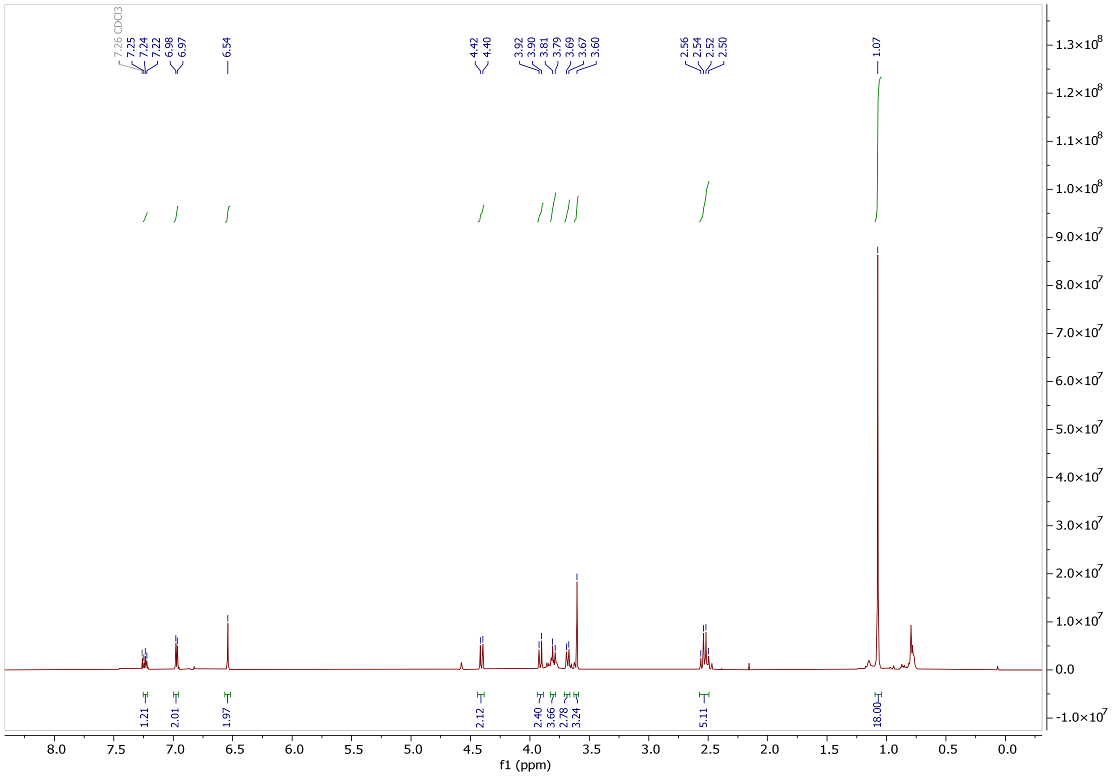

Figure S4. ^{1}H NMR of **L2** in $CDCl_3$.

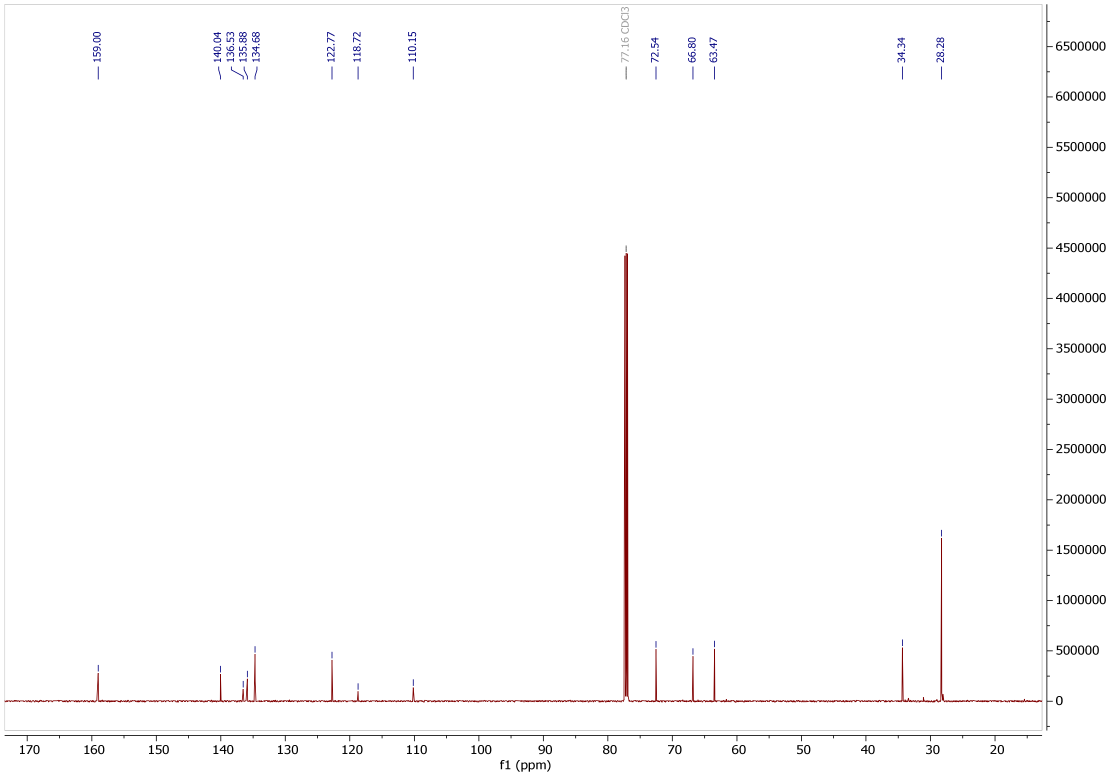

Figure S5. ^{13}C NMR of **L2** in $CDCl_3$.

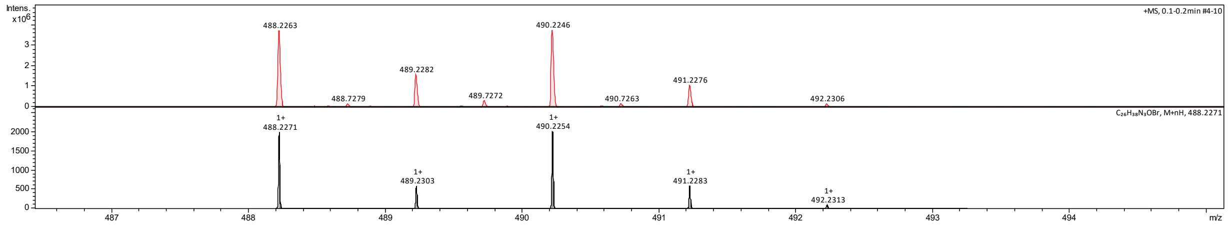

Figure S6. Experimental (top) and simulated (bottom) ESI-MS spectra of **L2** in MeCN.

Synthesis of L3

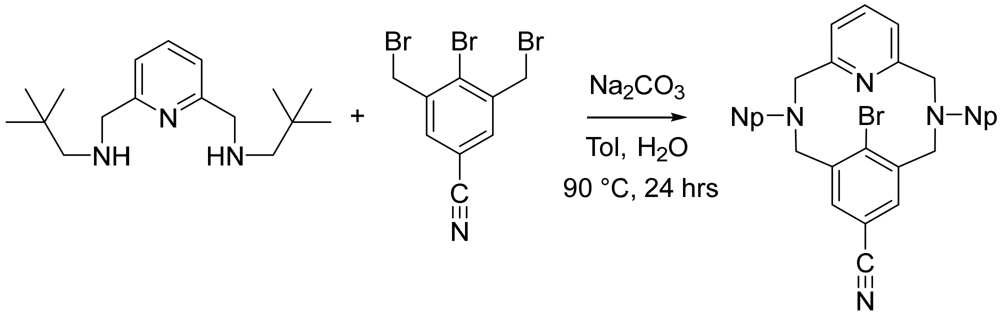

A 250 mL three-neck round bottom flask equipped with a reflux condenser, an addition funnel, and a magnetic stirring bar was charged with a solution of N,N'-(pyridine-2,6-diylbis(methylene))bis(2,2-dimethylpropan-1-amine) (0.52 g, 1.88 mmol, 1 equiv) in 100 mL of toluene and 60 mL of 40 % aqueous solution of Na_2CO_3. The stirred reaction mixture was pre-heated in an oil bath at 90 °C. A solution of 4-bromo-3,5-bis(bromomethyl)benzonitrile (0.69 g, 1.88 mmol, 1 equiv) in 20 mL toluene was added dropwise to the stirred reaction mixture at 90 °C over 2 hours. The resulting solution was heated at 90 °C under N_2 for an additional 12 hours. The reaction mixture was cooled down to RT, the organic layer was separated, and the solvent was removed in vacuo to generate a yellowish oil mixture. The yellowish yellow oil was extracted with pentane (3×5 mL). The solution was concentrated to ~5 mL and stored at -20 °C overnight. The solution was filtered to remove any precipitate. The solvent was removed to yield yellow oil. (yield: 800 mg, 74%).

^{1}H NMR (600 MHz, $CDCl_3$) δ 7.36 (t, J = 7.6 Hz, 1H), 7.21 (s, 2H), 6.98 (d, J = 7.7 Hz, 2H), 4.46 (d, J = 12.9 Hz, 2H), 3.90 (d, J = 13.0 Hz, 2H), 3.83 (d, J = 14.6 Hz, 2H), 3.75 (d, J = 13.6 Hz, 2H), 2.57 (q, J = 13.6 Hz, 4H), 1.09 (s, 18H).

^{13}C NMR (151 MHz, $CDCl_3$) δ 159.00, 140.04, 136.53, 135.88, 134.68, 122.77, 118.72, 110.15, 72.54, 66.80, 63.47, 34.34, 28.28.

ESI-MS of **L3** in MeCN: m/z 483.2123 (calcd for [**L3**·H]$^+$, $C_{26}H_{36}BrN_4$, m/z 483.2118).

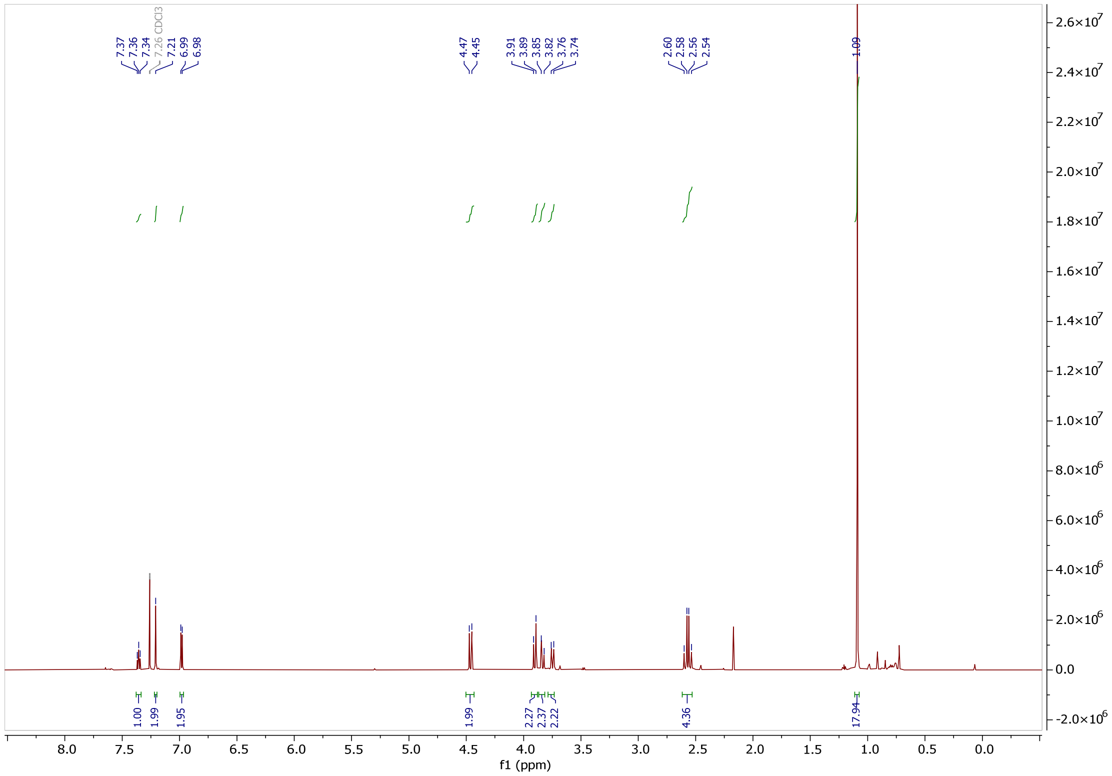

Figure S7. ^{1}H NMR of **L3** in $CDCl_3$.

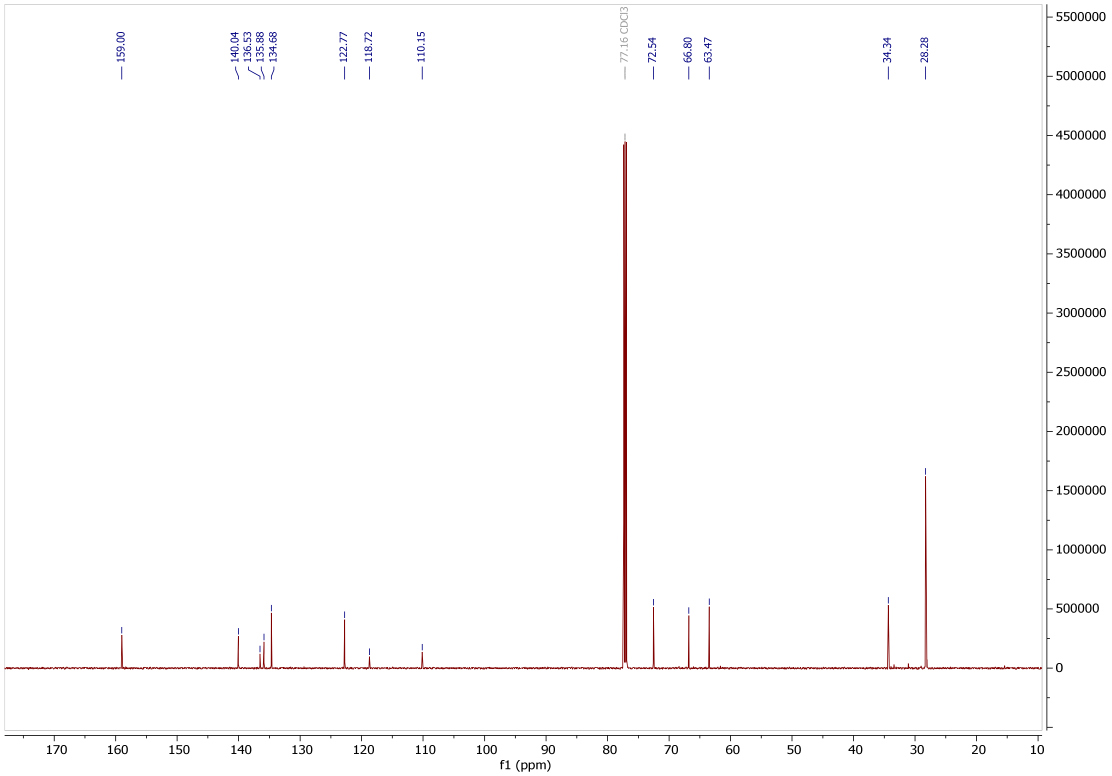

Figure S8. ^{13}C NMR of **L3** in $CDCl_3$.

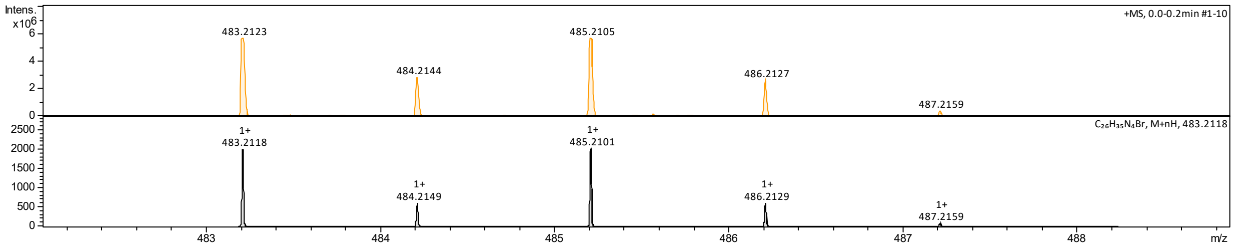

Figure S9. Experimental (top) and simulated (bottom) ESI-MS spectra of **L3** in MeCN.

Synthesis of L6

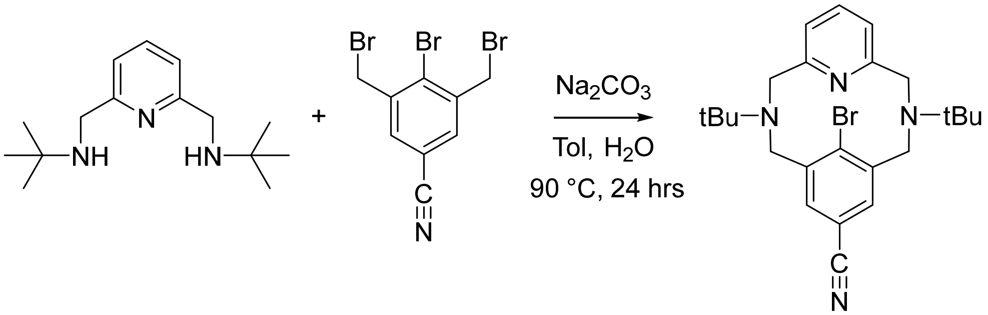

A 250 mL three-neck round bottom flask equipped with a reflux condenser, an addition funnel, and a magnetic stirring bar was charged with a solution of N,N'-(pyridine-2,6-diylbis(methylene))bis(2-methylpropan-2-amine) (0.60 g, 2.40 mmol, 1 equiv) in 100 mL of toluene and 60 mL of 40 % aqueous solution of Na_2CO_3. The stirred reaction mixture was pre-heated in an oil bath at 90 °C. A solution of 4-bromo-3,5-bis(bromomethyl)benzonitrile (0.89 g, 2.40 mmol, 1 equiv) in 20 mL toluene was added dropwise to the stirred reaction mixture at 90 °C over 2 hours. The resulting solution was heated at 95 °C under N_2 for an additional 12 hours. The reaction mixture was cooled down to RT, the organic layer was separated and dried, and the solvent was removed in vacuo to generate a white solid. The white solid was dissolved into 4 mL of DCM and layered with 16 mL of pentane. White crystals, which were identified as the product, were grown over 2 hours. The left mother solution can be dried to set up recrystallization to generate more products. (yield: 880 mg, 82%).

^{1}H NMR (500 MHz, $cdcl_3$) δ 7.28 (t, *J* = 7.6 Hz, 1H), 7.05 (s, 2H), 6.78 (d, *J* = 7.6 Hz, 2H), 4.16 (dd, *J* = 13.3, 6.5 Hz, 4H), 4.05 (d, *J* = 13.1 Hz, 2H), 3.49 (d, *J* = 13.4 Hz, 2H), 1.32 (s, 18H).

^{13}C NMR (126 MHz, c_6d_6) δ 159.89, 141.04, 135.52, 134.42, 121.89, 120.72, 118.95, 109.81, 57.03, 56.27, 54.30, 27.85.

ESI-MS of **L6** in MeCN: m/z 455.1801 (calcd for [**L6**·H]$^+$, $C_{24}H_{32}BrN_4$, m/z 455.1805).

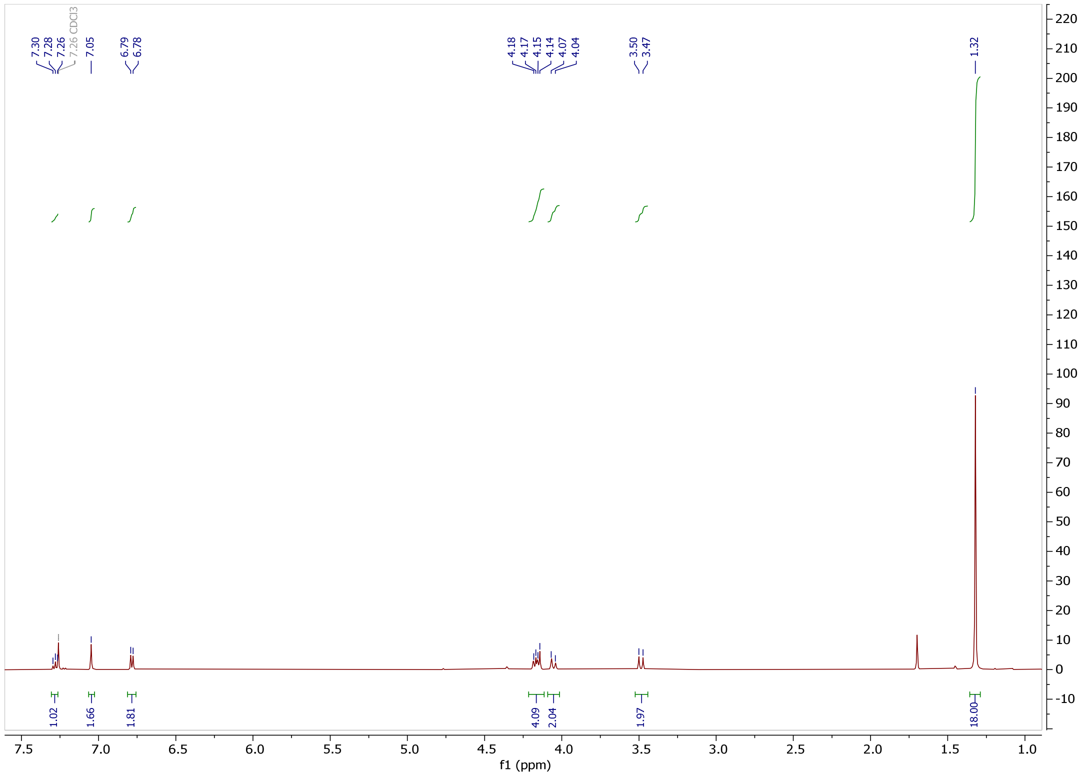

Figure S10. ^{1}H NMR of **L6** in $CDCl_3$.

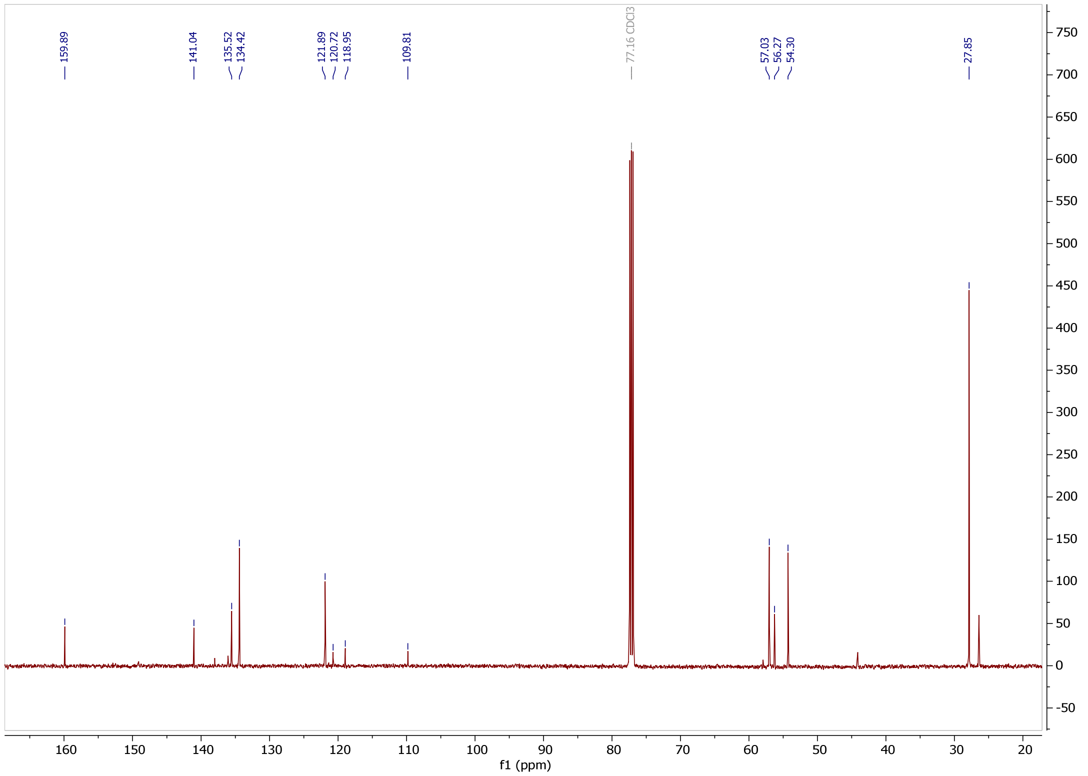

Figure S11. ^{13}C NMR of **L6** in $CDCl_3$.

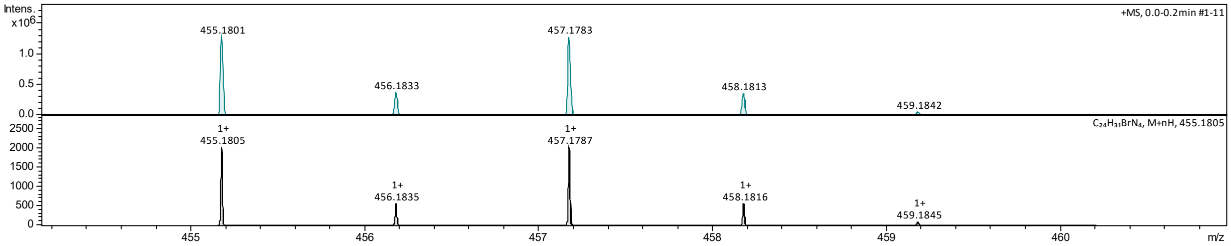

Figure S12. Experimental (top) and simulated (bottom) ESI-MS spectra of **L6** in MeCN.

Synthesis of [(L1)Pd^{III}(MeCN)Br]PF_6 or BF_4 (1)

To a solution of **7** (12.1 mg, 0.019 mmol, 1 equiv) in 0.5 mL of MeCN, **L1** (17.5 mg, 0.038 mmol, 2 equiv) in 0.5 mL of THF was added. The reaction was stirred for 30 min at room temperature and turned green from orange. Dark green crystals of [(**L1**)Pd^{III}(MeCN)Br]BF_4 were grown by layering with Et_2O at room temperature overnight (yield: 21.6 mg, 82%).

ESI-MS of [(**L1**)Pd^{III}(MeCN)Br]PF_6 in CH_3CN: m/z 563.1117, (calcd for [(**L1**)Pd^{III}Br]$^+$, $C_{25}H_{36}PdBrN_3$, m/z 563.1126).

^{19}F NMR (δ ppm, CD_3CN, 293 K, 300 MHz): -72.5 (d, 6F, PF_6).

Evans' method (CD_3CN): μ_{eff} = 1.72 B.M.

UV-vis (CH_3CN) λ_{max} (nm) (ε [$M^{-1}cm^{-1}$]): 452(1064), 632(724).

Elemental analysis of [(L1)Pd^{III}(MeCN)Br]PF_6: calcd for $C_{27}H_{39}PBrF_6N_4Pd$: C, 43.19; H, 5.24; N, 7.46; found: C, 43.62; H, 5.06; N, 7.19.

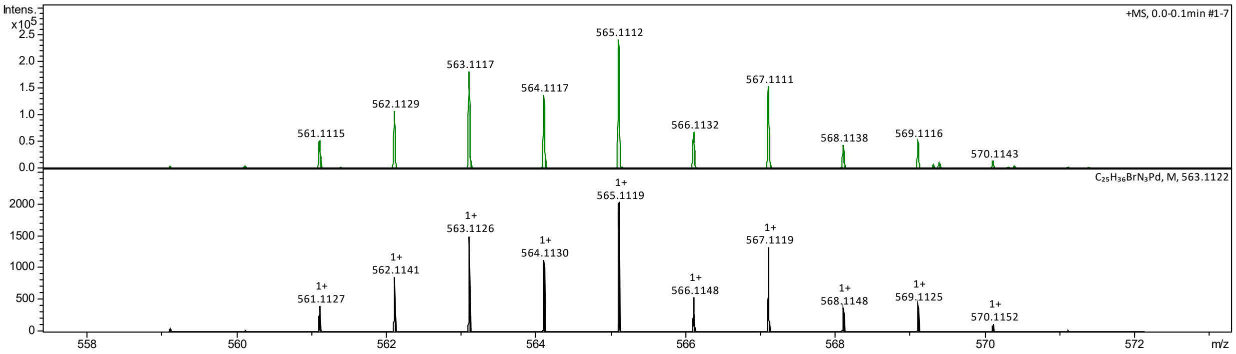

Figure S13. Experimental (top) and simulated (bottom) ESI-MS spectra of complex **1** in MeCN**.**

Synthesis of [(L2)Pd^{III}(MeCN)Br]BF_4 (2)

To a solution of **7** (19.1 mg, 0.030 mmol, 1 equiv) in 0.5 mL of MeCN, **L2** (29.5 mg, 0.060 mmol, 2 equiv) in 0.5 mL of THF was added. The reaction was stirred for 30 min at room temperature and turned yellowish green from orange. A filtration was applied to remove any precipitate. The filtered solution was concentrated to ~ 0.7 mL and was added with 10 mL of Et_2O. The product was isolated as a green powder. (yield: 35 mg, 80%).

ESI-MS of [(**L2**)Pd^{III}(MeCN)Br]BF_4 in CH_3CN: m/z 593.1125, (calcd for [(**L2**)Pd^{III}Br]$^+$, $C_{26}H_{38}PdOBrN_3$, m/z 593.1132).
^{19}F NMR (δ ppm, CD_3CN, 293 K, 300 MHz): -151.3 (s, 4F, BF_4).
Evans' method (CD_3CN): μ_{eff} = 1.74 B.M.
UV-vis (CH_3CN) λ_{max} (nm) (ε [$M^{-1}cm^{-1}$]): 464(762), 632(453).

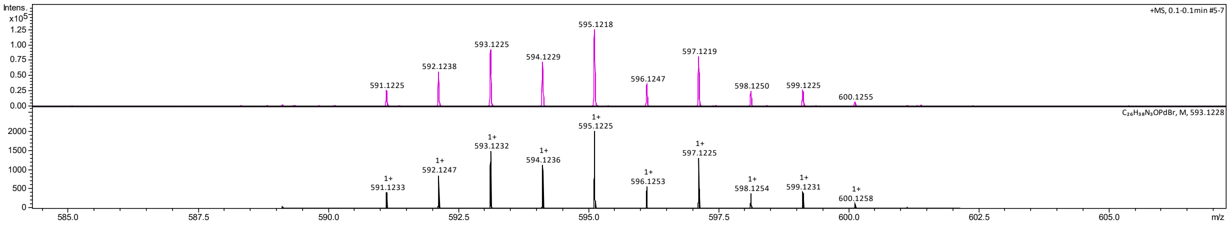

Figure S14. Experimental (top) and simulated (bottom) ESI-MS spectra of complex **2** in MeCN.

Synthesis of [(L3)Pd^{III}(MeCN)Br]BF_4 (3)

To a solution of **7** (22.0 mg, 0.035 mmol, 1 equiv) in 1.0 mL of MeCN, **L3** (33.5 mg, 0.070 mmol, 2 equiv) in 1.0 mL of THF was added. The reaction was stirred for 30 min at room temperature and turned green. A filtration was applied to remove any precipitate. The resulting solution was concentrated to 1 mL. 10 mL of Et_2O was added to this concentrated solution to precipitate the green product. The green solid was washed by Et_2O (3×1 mL) and dried under vacuum. (yield: 41.3 mg, 84%).
ESI-MS of [(**L3**)Pd^{III}(MeCN)Br]BF_4 in CH_3CN: m/z 588.1070, (calcd for [(**L3**)Pd^{III}Br]$^+$, $C_{26}H_{35}PdBrN_4$, m/z 588.1084).
^{19}F NMR (δ ppm, CD_3CN, 293 K, 300 MHz): -151.3 (s, 4F, BF_4).
Evans' method (CD_3CN): μ_{eff} = 1.79 B.M.
UV-vis (CH_3CN) λ_{max} (nm) (ε [$M^{-1}cm^{-1}$]): 458(1121), 632(732).

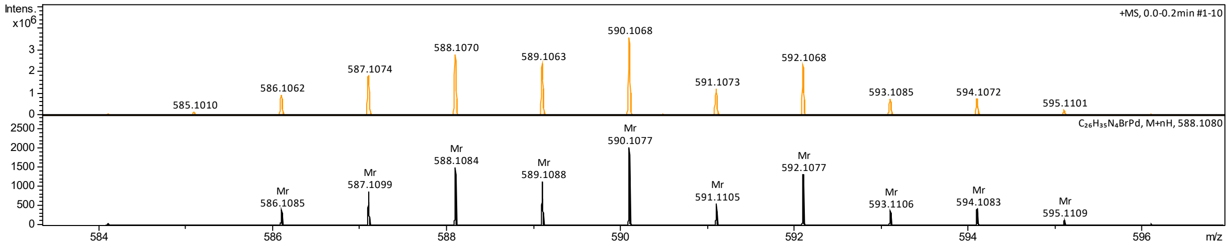

Figure S15. Experimental (top) and simulated (bottom) ESI-MS spectra of complex **3** in MeCN.

Synthesis of [(L4)Pd^{III}(MeCN)Br]BF_4 (4)

To a solution of **7** (17.3 mg, 0.027 mmol, 1 equiv) in 1.0 mL of MeCN, **L4** (23.7 mg, 0.055 mmol, 2 equiv) in 1.0 mL of THF was added. The reaction was stirred for 12 hours at room temperature

and turned green from orange. Green crystals of [(**L4**)Pd^{III}(MeCN)Br]PF_6 suitable for X-ray study were grown by layering with Et_2O using PF_6 as counter ion at room temperature overnight (yield: 28.7 mg, 79%).
ESI-MS of [(**L4**)Pd^{III}(MeCN)Br]PF_6 in CH_3CN: m/z 535.0805, (calcd for [(**L4**)$Pd^{III}Br]^+$, $C_{23}H_{32}PdBrN_3$, m/z 535.0812).
^{19}F NMR (δ ppm, CD_3CN, 293 K, 300 MHz): -72.5 (d, 6F, PF_6).
Evans' method (CD_3CN): μ_{eff} = 1.78 B.M.
UV-vis (CH_3CN) λ_{max} (nm) (ε [$M^{-1}cm^{-1}$]): 473(1500), 698(926).
Elemental analysis of [(**L4**)Pd^{III}(MeCN)Br]BF_4: calcd for $C_{25}H_{35}BBrF_4N_4Pd$: C, 45.17; H, 5.31; N, 8.43; found: C, 44.91; H, 5.11; N, 8.47.

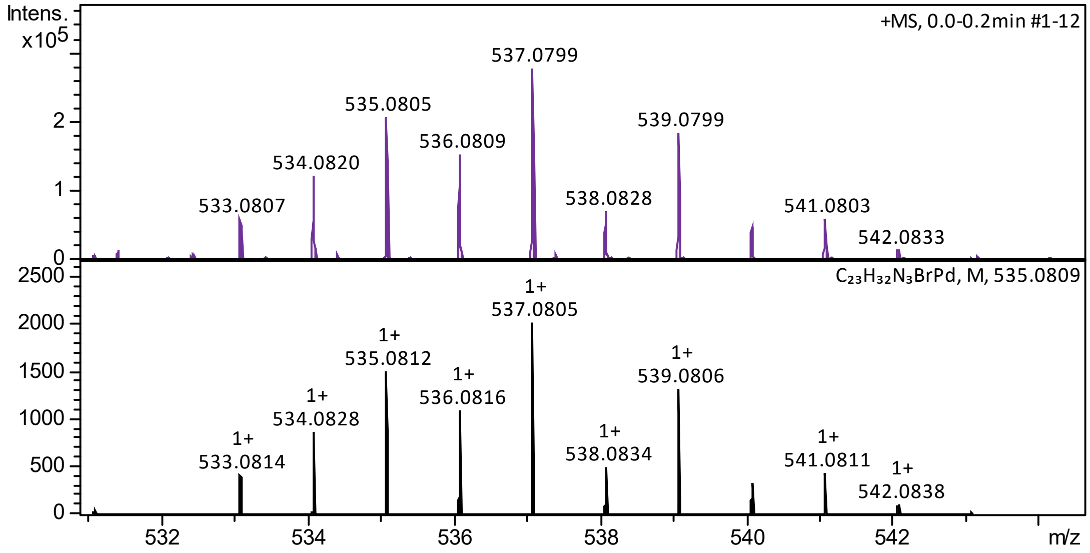

Figure S16. Experimental (top) and simulated (bottom) ESI-MS spectra of complex **4** in MeCN.

Synthesis of [(L5)Pd^{III}(MeCN)Br]BF_4 (5)

To a solution of **7** (8.3 mg, 0.013 mmol, 1 equiv) in 1.0 mL of MeCN, **L5** (12.7 mg, 0.026 mmol, 2 equiv) in 1.0 mL of THF was added. The reaction was stirred for 12 hours at room temperature and turned yellowish green from orange. A filtration was applied to remove any precipitate. The resulting solution was concentrated to 0.5 mL. 10 mL of Et_2O was added to this concentrated solution to precipitate the yellowish green product. The green solid was washed by Et_2O (3×1 mL) and dried under vacuum. (yield: 12.3 mg, 67%).

ESI-MS of [(**L5**)Pd^{III}(MeCN)Br]BF_4 in CH_3CN: m/z 565.0922, (calcd for [(**L5**)$Pd^{III}Br]^+$, $C_{24}H_{34}PdOBrN_3$, m/z 565.0918).
^{19}F NMR (δ ppm, CD_3CN, 293 K, 300 MHz): -151.3 (s, 4F, BF_4).
Evans' method (CD_3CN): μ_{eff} = 1.72 B.M.
UV-vis (CH_3CN) λ_{max} (nm) (ε [$M^{-1}cm^{-1}$]): 480(1085), 693(805).

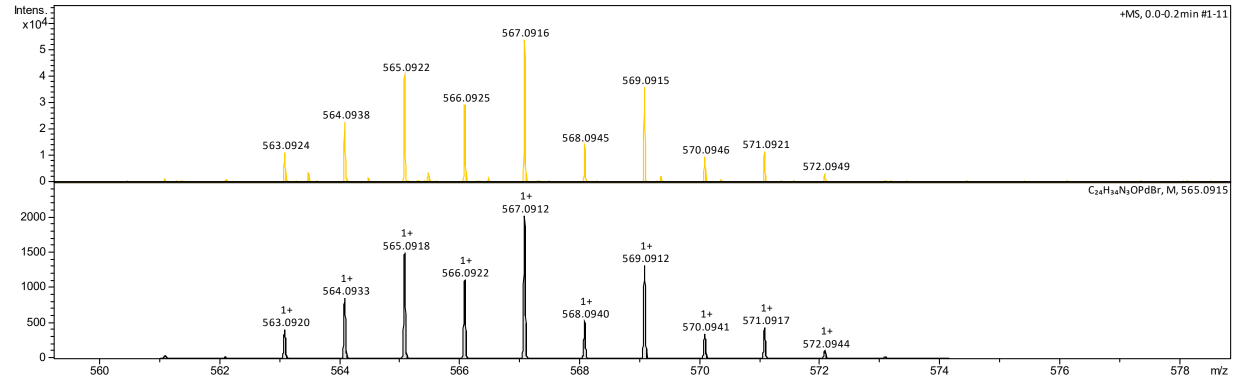

Figure S17. Experimental (top) and simulated (bottom) ESI-MS spectra of complex **5** in MeCN.

Synthesis of [(L6)Pd^{III}(MeCN)Br]BF_4 (6)

To a solution of **7** (19.3 mg, 0.031 mmol, 1 equiv) in 1.0 mL of MeCN, **L6** (27.7 mg, 0.062 mmol, 2 equiv) in 1.0 mL of THF was added. The reaction was stirred for 12 hours at room temperature and turned yellowish green from orange. A filtration was applied to remove any precipitate. The resulting solution was concentrated to 1 mL. 10 mL of Et_2O was added to this concentrated solution to precipitate the yellowish green product. The green solid was washed by Et_2O (3×1 mL) and dried under vacuum. (yield: 25.7 mg, 61%).

ESI-MS of [(**L6**)Pd^{III}(MeCN)Br]BF_4 in CH_3CN: m/z 560.0761, (calcd for [(**L6**)Pd^{III}Br]$^+$, $C_{24}H_{31}PdBrN_4$, m/z 560.0765).

^{19}F NMR (δ ppm, CD_3CN, 293 K, 300 MHz): -151.3 (s, 4F, BF_4).

Evans' method (CD_3CN): μ_{eff} = 1.71 B.M.

UV-vis (CH_3CN) λ_{max} (nm) (ε [$M^{-1}cm^{-1}$]): 468(1186), 698(854).

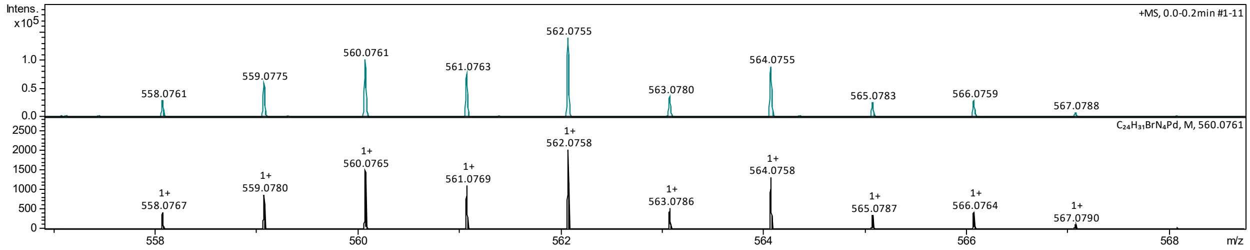

Figure S18. Experimental (top) and simulated (bottom) ESI-MS spectra of complex **6** in MeCN.

Synthesis of [Pd^{I}(MeCN)$_3$]$_2$(BF_4)$_2$ (7)

The Pd^{I} precursor was synthesized according to a slightly modified literature procedure.[7] To a solution of [Pd(MeCN)$_4$](BF_4)$_2$ (52.1 mg, 0.117 mmol, 2 equiv) in 0.5 mL of MeCN, $Pd_2(dba)_3$ (54.0 mg, 0.059 mmol, 1 equiv) in 0.5 mL of CH_2Cl_2 was added. The reaction was stirred for 20 min at room temperature. Then, the reaction mixture was filtered to remove any precipitate. The filtered reaction was concentrated to 0.5 mL under vacuum. To this concentrated reaction, 10 mL of Et_2O was added. Orange precipitate was generated immediately. The orange precipitate was washed with Et_2O (3×1 mL) (Yield: 69.0 mg, 92%).

III. Characterization Data

Cyclic Voltammetry Data

Electrochemical grade n-Bu_4NPF_6 was used as the supporting electrolyte. Cyclic voltammetry measurements were carried out in a glovebox under a dinitrogen atmosphere in a one-compartment cell using a CH Instruments electrochemical analyzer. A glassy carbon electrode and platinum wire were used as the working and auxiliary electrodes, respectively. The pseudo reference electrode was an Ag/AgCl wire. The reference electrode was calibrated vs Cp_2Fe.

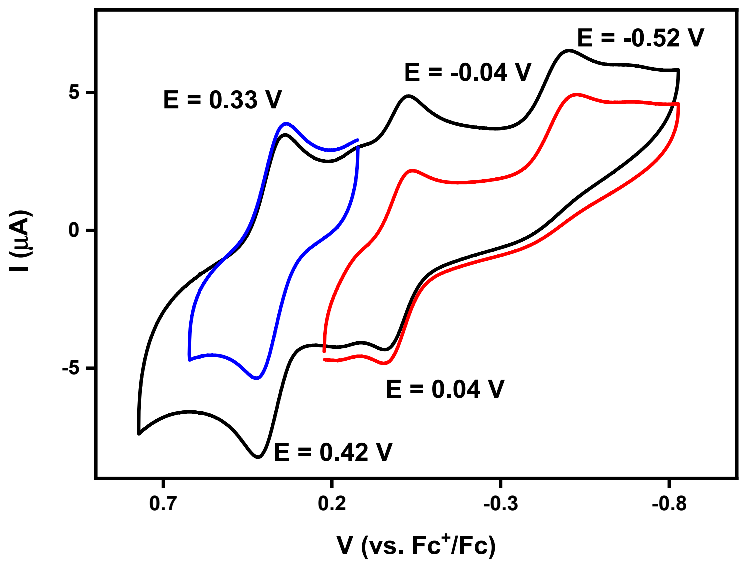

Figure S19. Cyclic voltammograms of complex **1** in 0.1 M n-Bu_4NBF_4/MeCN (100 mV/s scan rate, the black and colored traces represent wide and narrow potential range CVs, respectively).

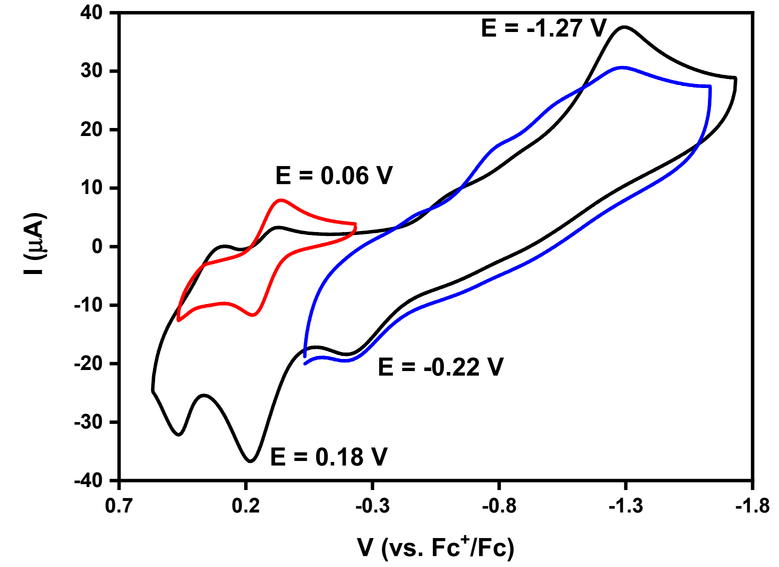

Figure S20. Cyclic voltammograms of complex **2** in 0.1 M n-Bu_4NBF_4/MeCN (100 mV/s scan rate, the black and colored traces represent wide and narrow potential range CVs, respectively).

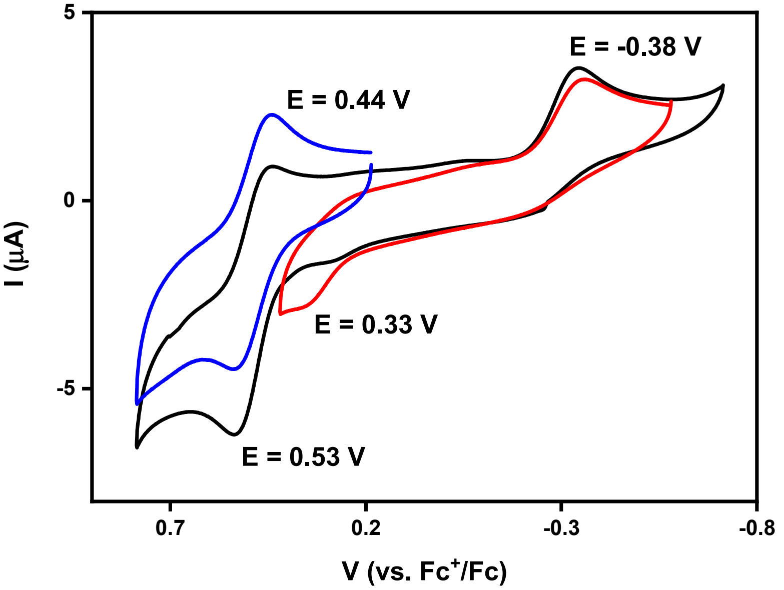

Figure S21. Cyclic voltammograms of complex **3** in 0.1 M *n*-Bu_4NBF_4/MeCN (100 mV/s scan rate, the black and colored traces represent wide and narrow potential range CVs, respectively).

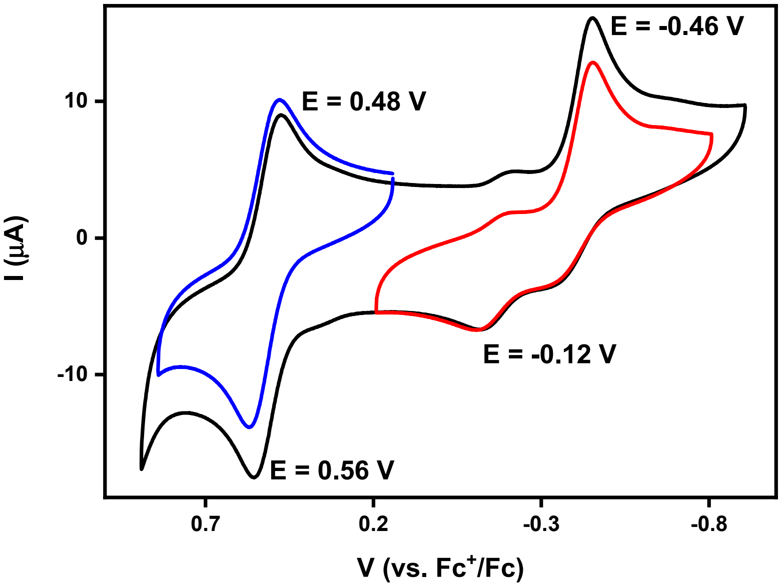

Figure S22. Cyclic voltammograms of complex **4** in 0.1 M *n*-Bu_4NBF_4/MeCN (100 mV/s scan rate, the black and colored traces represent wide and narrow potential range CVs, respectively).

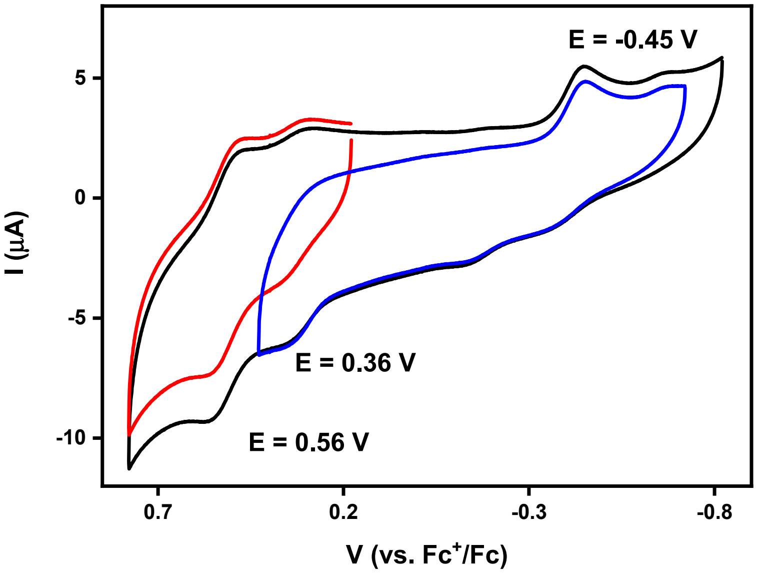

Figure S23. Cyclic voltammograms of complex **5** in 0.1 M *n*-Bu_4NBF_4/MeCN (100 mV/s scan rate, the black and colored traces represent wide and narrow potential range CVs, respectively).

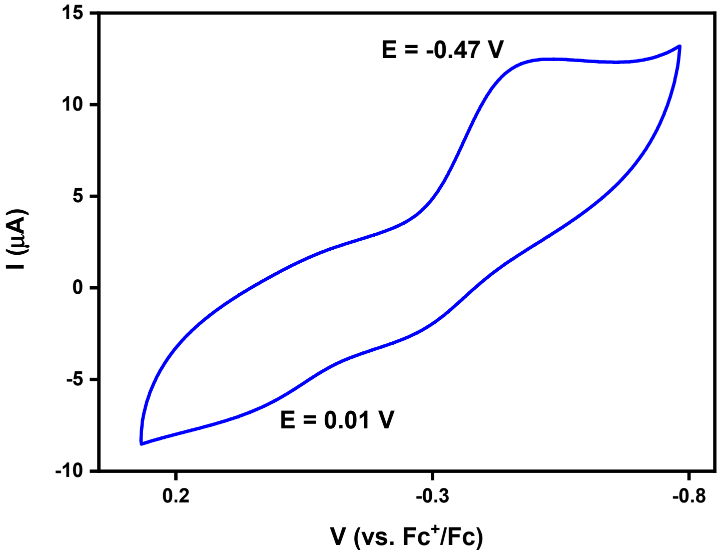

Figure S24. Cyclic voltammograms of complex **6** in 0.1 M *n*-Bu_4NBF_4/MeCN (100 mV/s scan rate).

UV-Vis Data

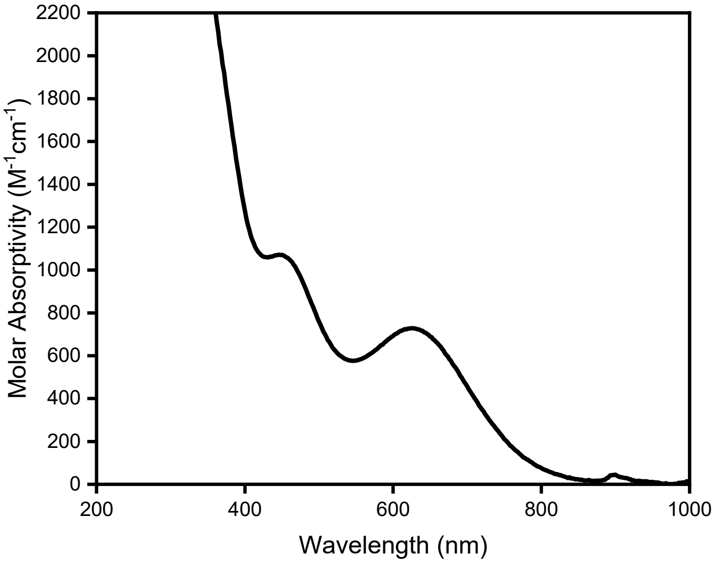

Figure S25. UV-Vis spectrum of complex **1** in a 1:1 mixture of MeCN:THF at room temperature.

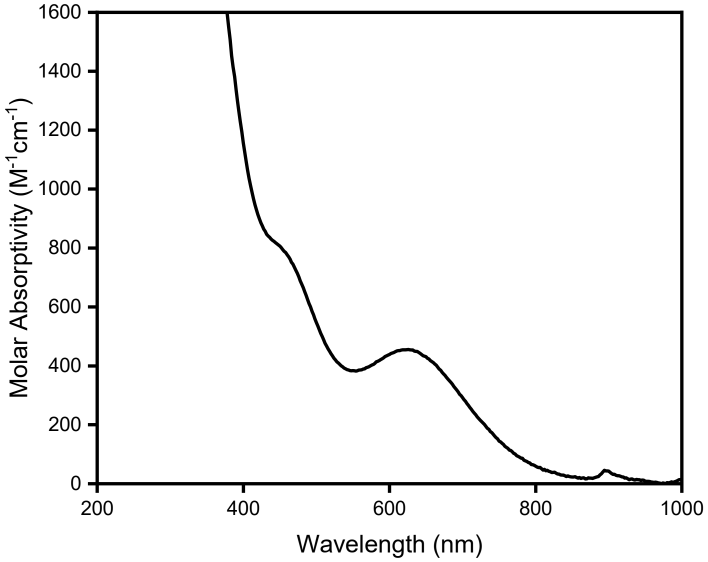

Figure S26. UV-Vis spectrum of complex **2** in a 1:1 mixture of MeCN:THF at room temperature.

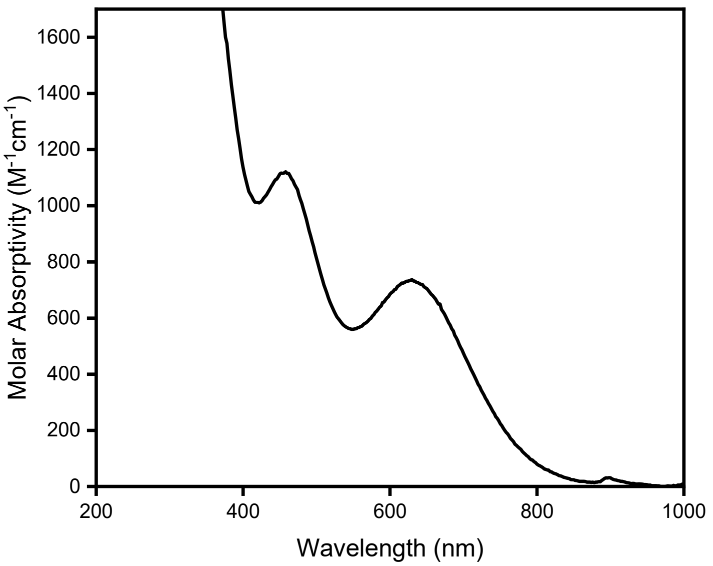

Figure S27. UV-Vis spectrum of complex **3** in a 1:1 mixture of MeCN:THF at room temperature.

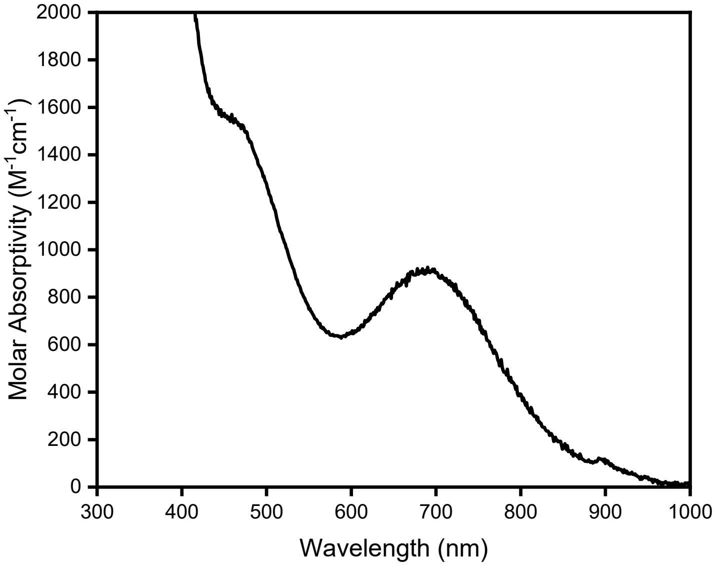

Figure S28. UV-Vis spectrum of complex **4** in a 1:1 mixture of MeCN:THF at room temperature.

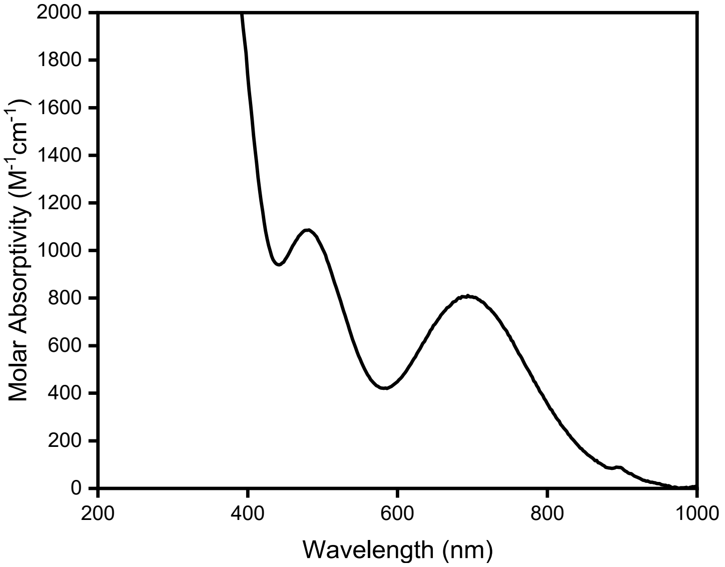

Figure S29. UV-Vis spectrum of complex **5** in a 1:1 mixture of MeCN:THF at room temperature.

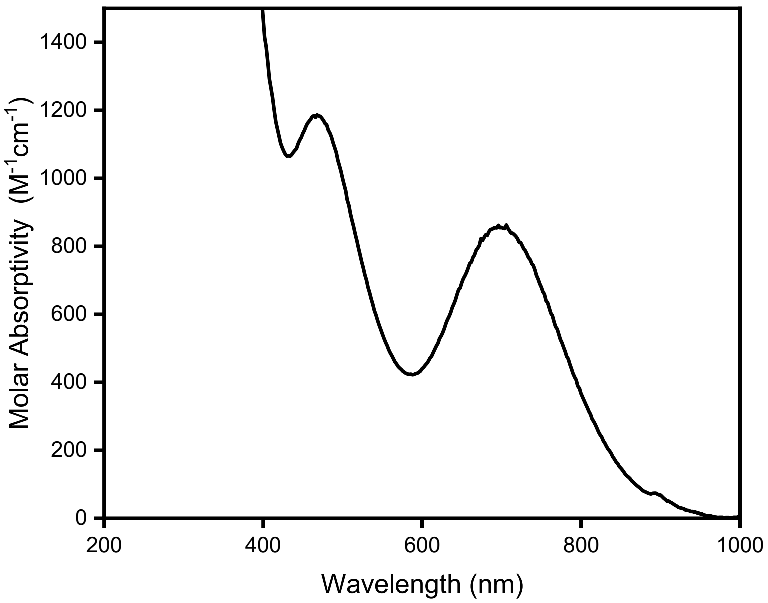

Figure S30. UV-Vis spectrum of **6** in a 1:1 mixture of MeCN:THF at room temperature.

Initial Rate Determinations

Initial rates were determined by using the following method (with exception to the formation of **1**, which was determined by stopped-flow UV-Vis at room temperature):

In a glovebox, 7.6 mg of Pd^{I} dimer **7** (0.012 mmol, or 15.2 mg for 2 equiv samples) was dissolved in 5 mL of a 1:1 mixture of MeCN:THF (2.4 mM). Separately, either 1 equiv ligand relative to the [Pd] concentration (i.e., 0.024 mmol, twice the concentration of dimer **7**), or 2 equiv of ligand (0.048 mmol), was dissolved in a 10 mL stock of a 1:1 mixture of MeCN:THF (2.4 mM). 2 mL of the ligand stock was transferred to a quartz cuvette and sealed with a septum and stir bar. 1 mL of the **7** solution was taken up in an airtight syringe, and the septum of the cuvette was punctured but not dispensed. The apparatus was removed from the glovebox and transferred to a UV-Vis sample holder situated over a stir plate. Under stirring, the solution of **7** was injected rapidly into the ligand solution, and the UV-Vis trace was measured over time. Curves used for initial rate determination were generated by taking the absorbance at 632 nm for ligands **L1-L3** or 698 nm for ligands **L4-L6** and subtracting the baseline for each time point. The initial rise for each plot was fitted linearly *via* a zero-order fitting, and the relative ratios for each ligand were used to determine reaction orders. All data was recorded in triplicate and errors are reported as 1 standard deviation.

For the reaction between **7** and **L1**, stopped-flow data was taken at room temperature for ligand:[Pd] ratios of 0.5:0.5, 1:1, and 2:1 to avoid instrument saturation. The relative dilution factors were calibrated to match those of the UV-Vis samples for ligands **L2-L6**. All data was recorded in triplicate and errors are reported as 1 standard deviation.

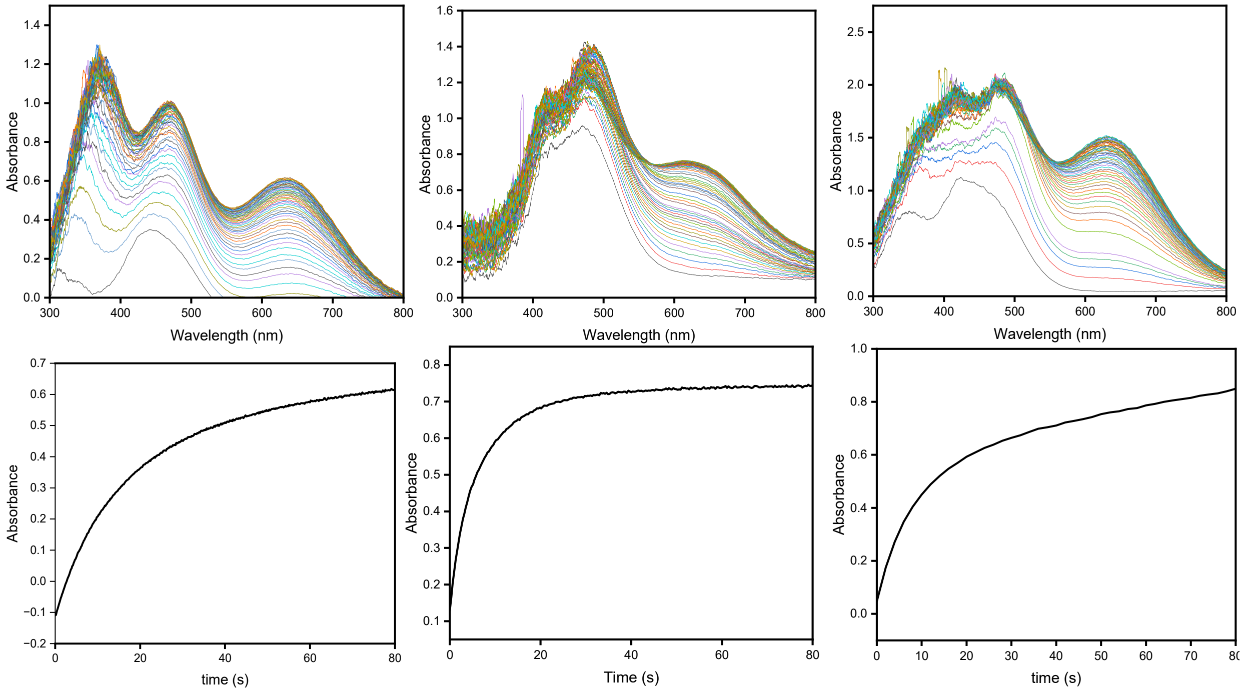

Figure S31. Stopped-flow UV-Vis data for the formation of complex **1** from **7** and **L1** in a 1:1 mixture of MeCN:THF at room temperature. (left top and bottom) UV-Vis plot and 632 nm trace

at the 0.5:0.5 **L1**:[Pd] ratio, (middle and bottom) UV-Vis plot and 632 nm trace at the 1:1 **L1**:[Pd] ratio, (right top and bottom) UV-Vis plot and 632 nm trace at the 2:1 **L1**: [Pd] ratio.

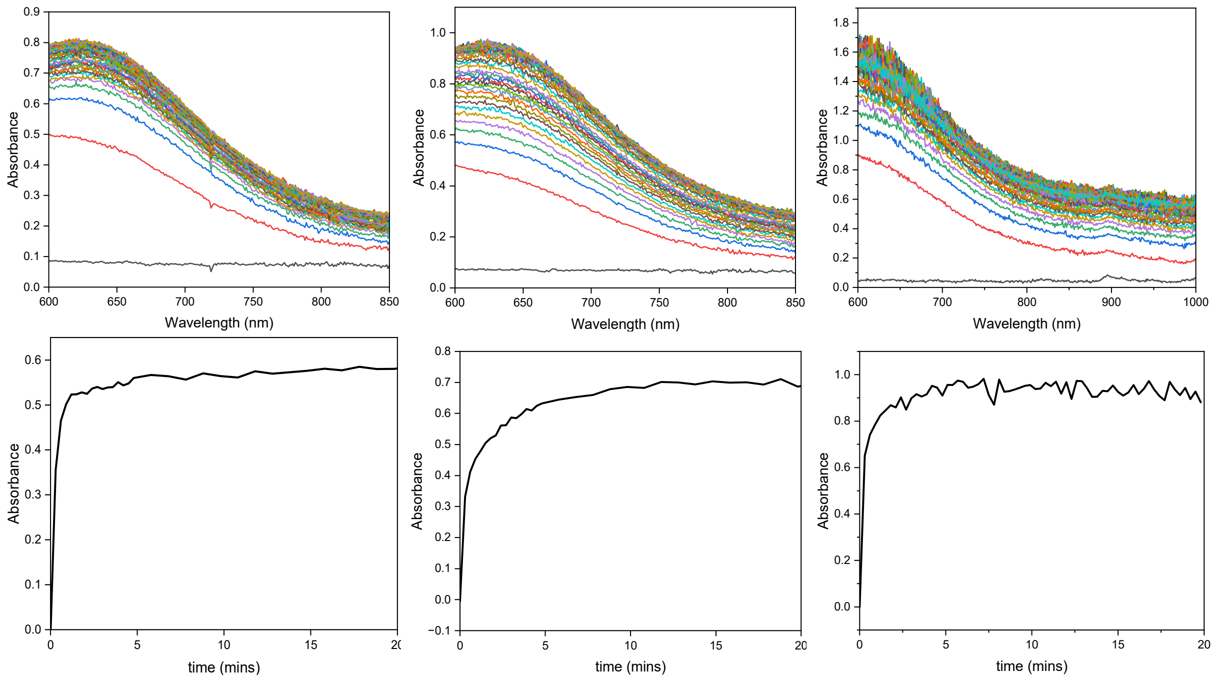

Figure S32. UV-Vis data for the formation of complex **2** from **7** and **L2** in a 1:1 mixture of MeCN:THF at room temperature. (left top and bottom) UV-Vis plot and 632 nm trace at the 1:1 **L2**:[Pd] ratio, (middle and bottom) UV-Vis plot and 632 nm trace at the 2:1 **L2**:[P] ratio, (right top and bottom) UV-Vis plot and 632 nm trace at the 2:2 **L2**:[Pd] ratio.

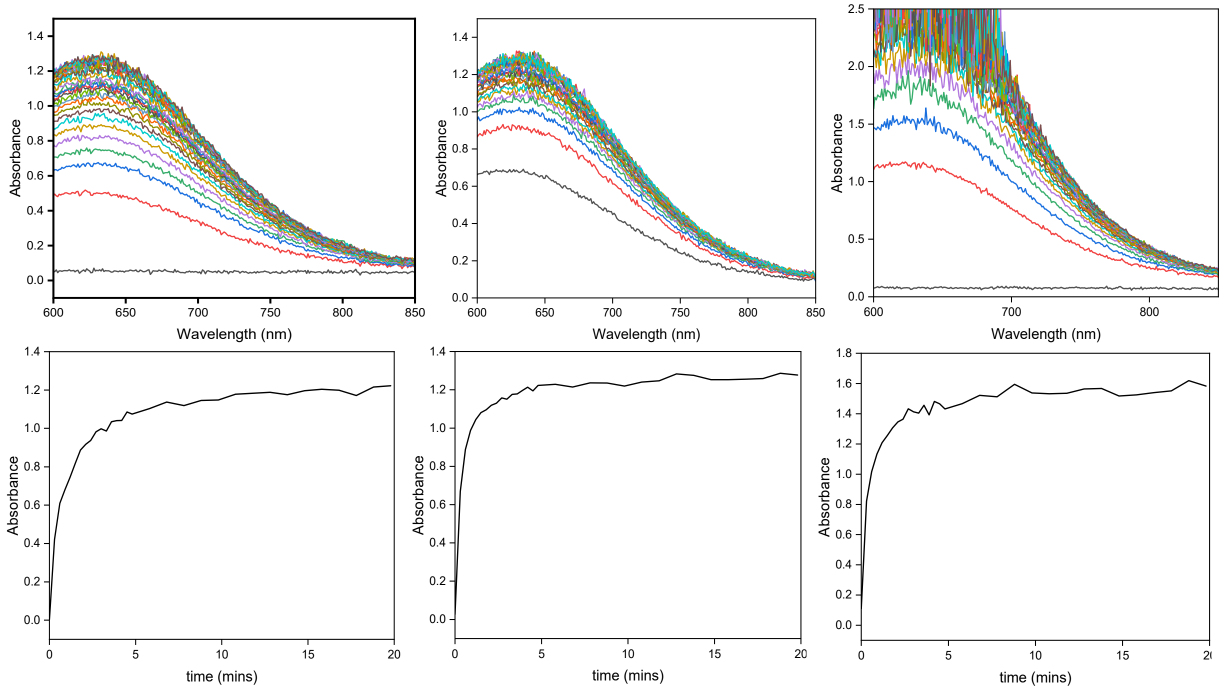

Figure S33. UV-Vis data for the formation of complex **3** from **7** and **L3** in a 1:1 mixture of MeCN:THF at room temperature. (left top and bottom) UV-Vis plot and 632 nm trace at the 1:1

L3:[Pd] ratio, (middle and bottom) UV-Vis plot and 632 nm trace at the 2:1 **L3**:[Pd] ratio, (right top and bottom) UV-Vis plot and 632 nm trace at the 2:2 **L3**:[Pd] ratio.

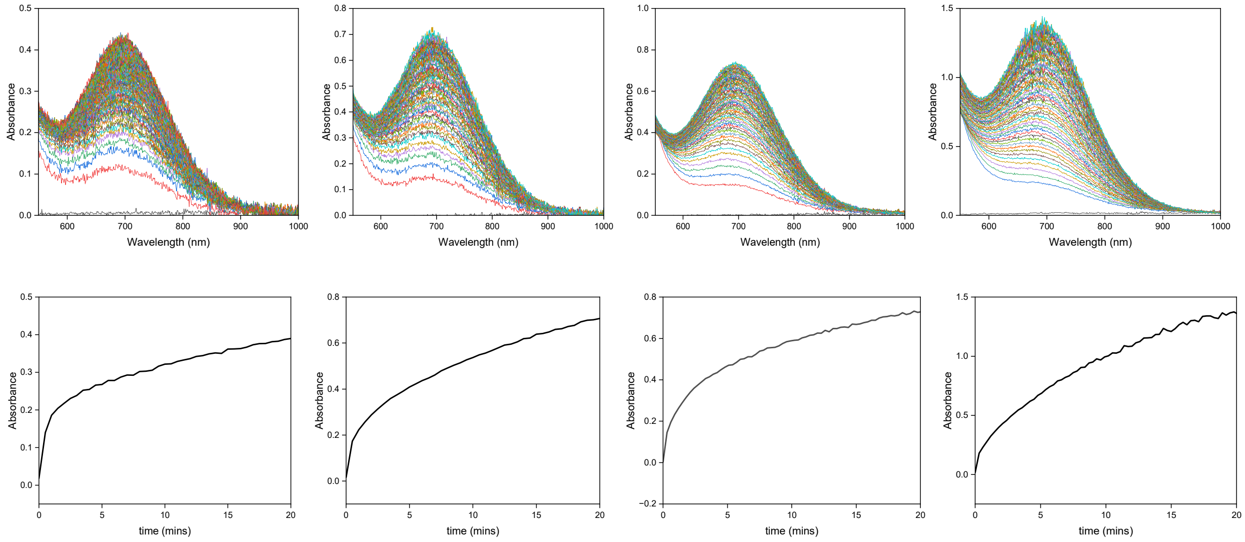

Figure S34. UV-Vis data for the formation of complex **4** from **7** and **L4** in a 1:1 mixture of MeCN:THF at room temperature. (left top and bottom) UV-Vis plot and 698 nm trace at the 0.5:0.5 **L4**:[Pd] ratio, (second from the left top and bottom) UV-Vis plot and 698 nm trace at the 1:1 **L4**:[Pd] ratio, (third from the left top and bottom) UV-Vis plot and 698 nm trace at the 2:1 **L4**:[Pd] ratio, (right top and bottom) UV-Vis plot and 698 nm trace at the 2:2 **L4**:[Pd] ratio.

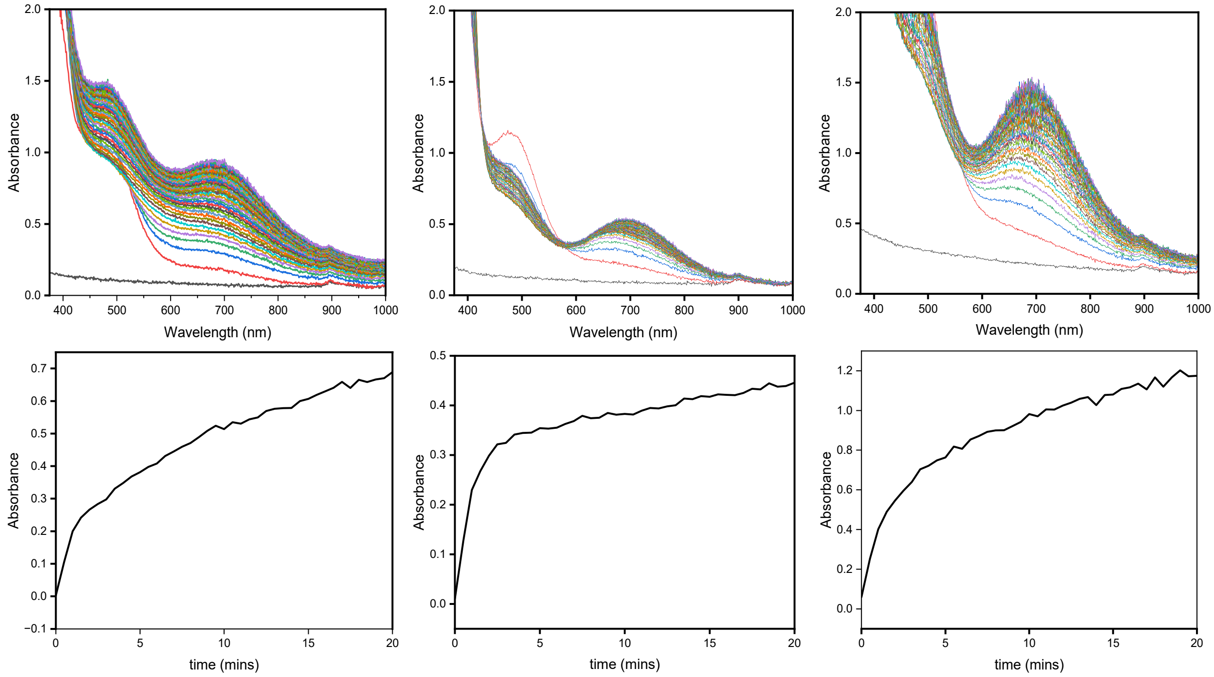

Figure S35. UV-Vis data for the formation of complex **5** from **7** and **L5** in a 1:1 mixture of MeCN:THF at room temperature. (left top and bottom) UV-Vis plot and 698 nm trace at the 1:1

L5:[Pd] ratio, (middle and bottom) UV-Vis plot and 698 nm trace at the 2:1 **L5**:[Pd] ratio, (right top and bottom) UV-Vis plot and 698 nm trace at the 2:2 **L5**:[Pd] ratio.

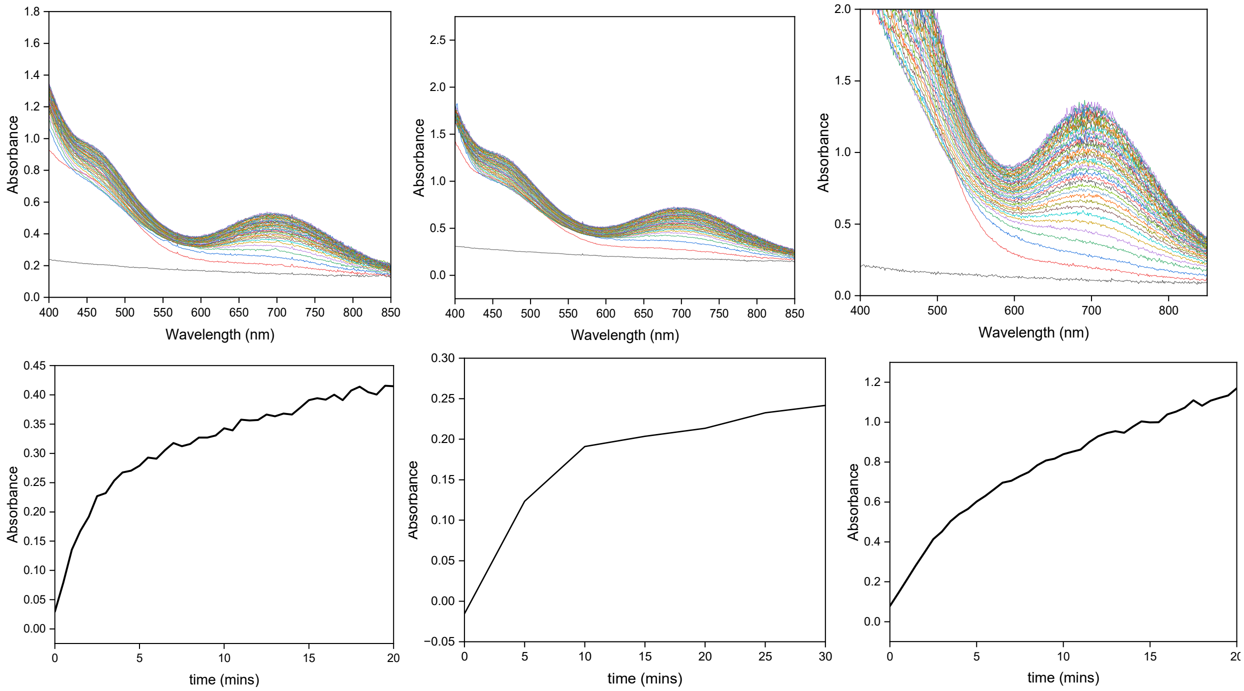

Figure S36. UV-Vis data for the formation of **6** from **7** and **L6** in a 1:1 mixture of MeCN:THF at room temperature. (left top and bottom) UV-Vis plot and 698 nm trace at the 1:1 **L6**:[Pd] ratio, (middle and bottom) UV-Vis plot and 698 nm trace at the 2:1 **L6**:[Pd] ratio, (right top and bottom) UV-Vis plot and 698 nm trace at the 2:2 **L6**:[Pd] ratio.

Table S1. Initial rates and reaction orders for the generation of complexes **1-6**; a 1:1 L:Pd ratio corresponds to a 1.6 mM solution in both Pd and ligand.[a] Use of a lower concentration of both Pd and ligand was necessary given the limitation of the stopped-flow instrument at high concentrations.

Ligand	Ratio L:Pd	Initial Rate (M/min)	Apparent Rate Equation
**L1**	0.5:0.5[a]	$1.20x10^{-3} \pm 0.04x10^{-3}$	$=k[Pd]^{0.5}[L]^{0}$
	1:1	$1.79x10^{-3} \pm 0.05x10^{-3}$	
	2:1	$1.93x10^{-3} \pm 0.16x10^{-3}$	
**L2**	1:1	$1.76x10^{-3} \pm 0.01x10^{-3}$	$=k[Pd]^{0.5}[L]^{0}$
	2:1	$1.50x10^{-3} \pm 0.05x10^{-3}$	
	2:2	$2.46x10^{-3} \pm 0.01x10^{-3}$	
**L3**	1:1	$1.35x10^{-3} \pm 0.07x10^{-3}$	$=k[Pd]^{0.5}[L]^{0}$
	2:1	$1.40x10^{-3} \pm 0.16x10^{-3}$	
	2:2	$2.09x10^{-3} \pm 0.04x10^{-3}$	
**L4**	0.5:0.5	$0.18x10^{-3} \pm 0.01x10^{-3}$	$=k[Pd]^{0.5}[L]^{0}$
	1:1	$0.25x10^{-3} \pm 0.03x10^{-3}$	
	2:1	$0.25x10^{-3} \pm 0.02x10^{-3}$	
	2:2	$0.36x10^{-3} \pm 0.02x10^{-3}$	
**L5**	1:1	$0.29x10^{-3} \pm 0.01x10^{-3}$	$=k[Pd]^{0.5}[L]^{0}$
	2:1	$0.31x10^{-3} \pm 0.04x10^{-3}$	
	2:2	$0.50x10^{-3} \pm 0.05x10^{-3}$	
**L6**	1:1	$0.09x10^{-3} \pm 0.01x10^{-3}$	$=k[Pd]^{0}[L]^{1}$
	2:1	$0.20x10^{-3} \pm 0.01x10^{-3}$	
	2:2	$0.19x10^{-3} \pm 0.02x10^{-3}$	

IV. Cryo Stopped-Flow UV-Vis Data

A typical stopped-flow UV-Vis experiment is described below:

In a glovebox, 7.6 mg of **7** (0.012 mmol or 15.2 mg for 2 equiv samples) was dissolved in 5 mL of a 1:1 mixture of MeCN:THF. Separately, 1 or 2 equiv ligand relative to [Pd] was dissolved in a 5 mL stock of a 1:1 mixture of MeCN:THF. These solutions were loaded into gas-tight syringes secured with syringe stoppers. 4 additional syringes were loaded with 5 mL of a 1:1 mixture of MeCN:THF and secured with syringe stoppers, 2 of which had the plungers removed and were secured shut with septa. The solutions containing ligand and complex were each inserted into the septa-secured syringes and all 4 syringe apparatuses were removed from the glovebox. Each syringe was attached to each of the 4 ports of the stopped-flow instrument, and the two lines bearing the complex and ligand solutions were placed under an Ar atmosphere with a balloon and needle. All lines were purged with the solvent mixture before the blank was measured. UV-Vis scans were obtained over the course of 80 seconds at 4 individual temperatures ranging from -10 °C to 20 °C. Traces were obtained from subtracting the baseline obtained at 798 nm from the relevant d-d absorption trace for each complex.

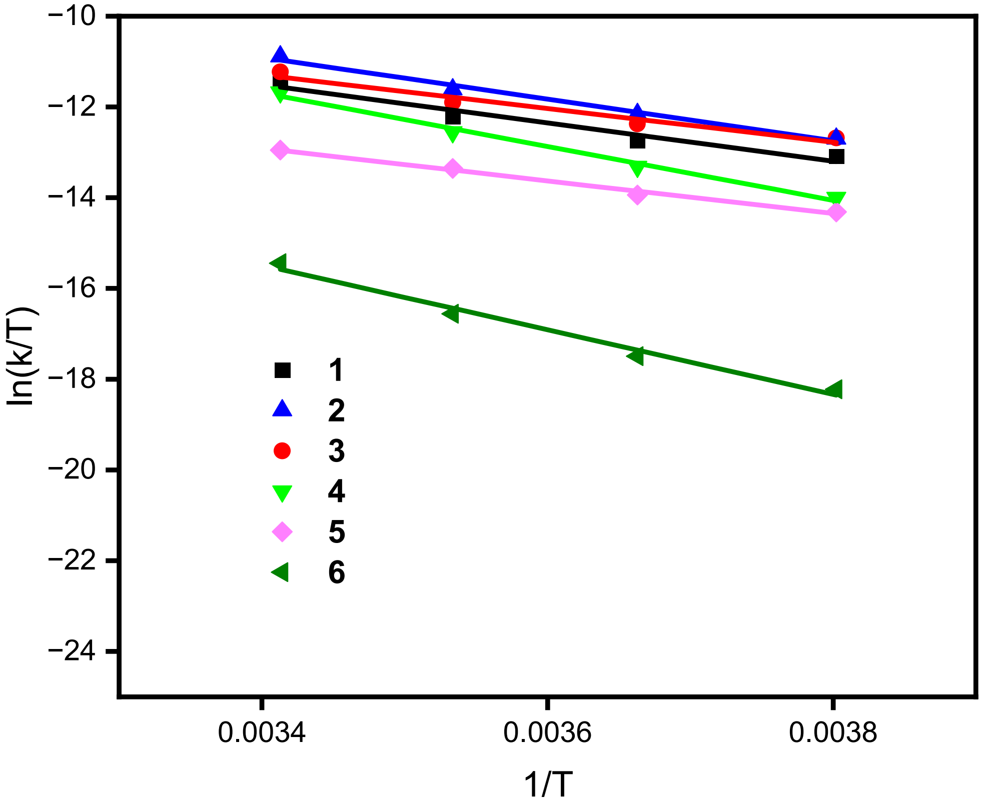

Figure S37. Eyring analysis for the generation of complexes **1-6** from the 1:1 reaction between **7** and the corresponding ligands (1 ligand per Pd ion) in a 1:1 mixture of MeCN:THF between -10 °C and 20 °C. Individual rates taken from the initial reaction rates.

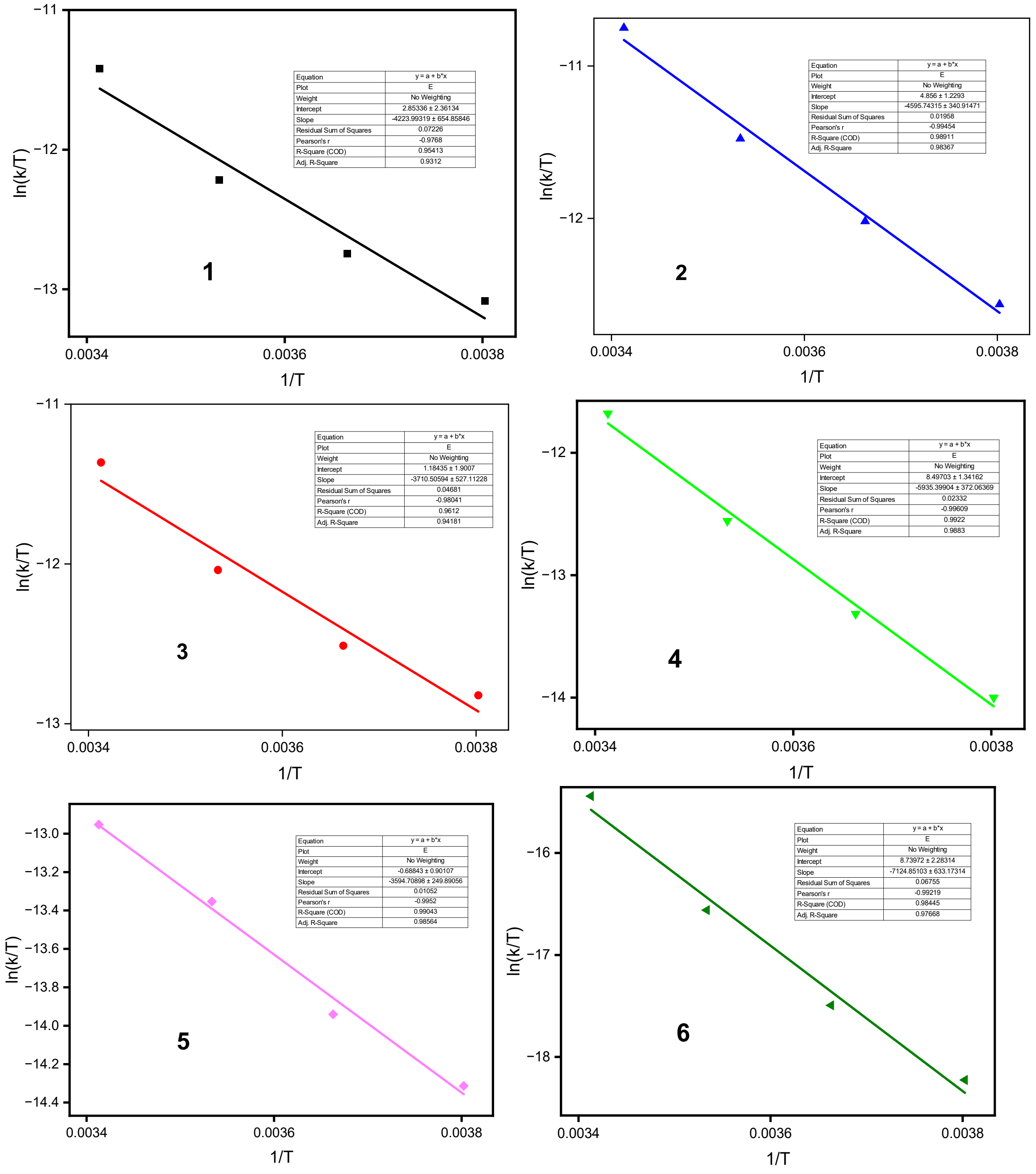

Figure S38. Individual fittings for complexes **1-6** extrapolated from Figure S37.

Table S2. Thermodynamic parameters derived from the Eyring analysis for the generation of complexes **1-6** from the 1:1 reaction between **7** and the corresponding ligands (1 ligand per Pd ion) in a 1:1 mixture of MeCN:THF.

Complex	$\Delta H^{\ddagger}$ (kcal/mol)	$\Delta S^{\ddagger}$ (cal/mol·K)	$\Delta G^{\ddagger}_{293}$ (kcal/mol)
**1**	8.4 ± 1.3	-41.5 ± 4.7	20.6 ± 1.9
**2**	9.1 ± 0.7	-37.6 ± 2.5	20.2 ± 1.0
**3**	7.4 ± 1.0	-44.9 ± 3.8	20.5 ± 1.5
**4**	11.8 ± 0.7	-30.7 ± 3.1	20.8 ± 0.9
**5**	7.1 ± 0.5	-48.6 ± 2.1	21.4 ± 0.8
**6**	14.2 ± 1.3	-29.8 ± 4.6	22.9 ± 1.9

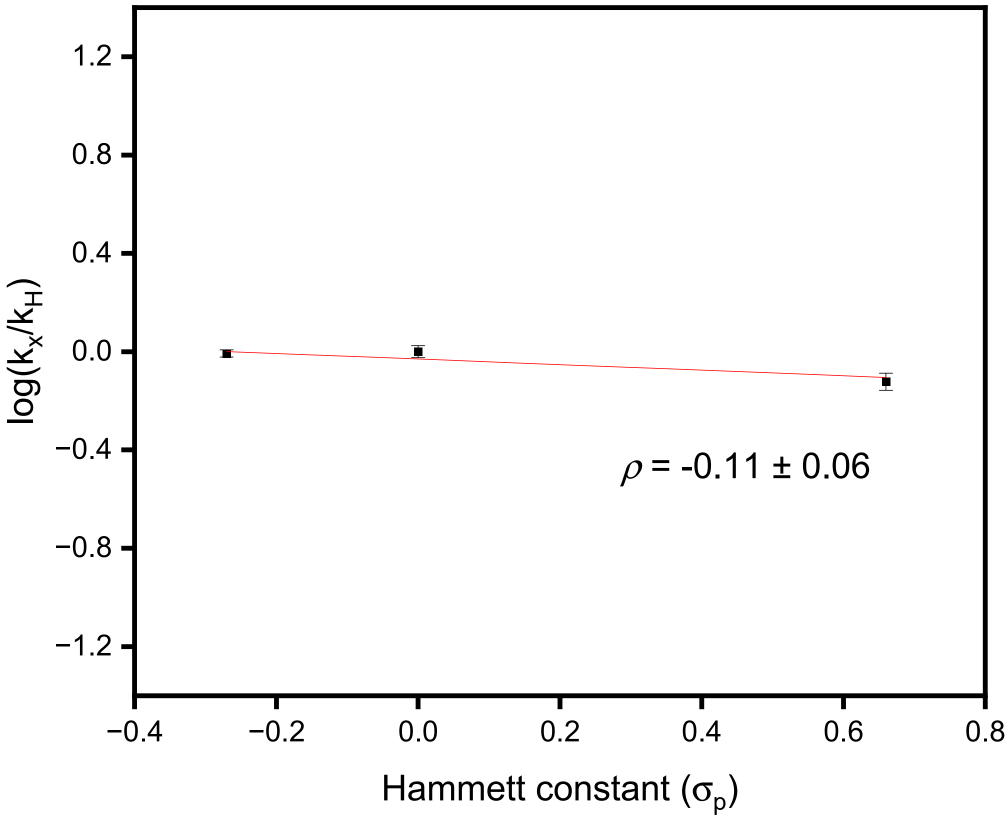

Figure S39. Hammett Analysis for the reactions between **7** and ligands **L1**-**L3** (1 ligand per Pd ion) in a 1:1 mixture of MeCN:THF at 20 °C. Rates taken from the initial reaction rates obtained in the stopped-flow experiments. Errors are represented as 90% confidence intervals of an average of 3 trials.

Due to the outlying nature of the reaction between **7** and **L6** in both reaction rate and reaction order, Hammett analysis was not performed across the series *tert*-butyl derived ligand frameworks. Doing so would lead to an artificially deflated value from which meaningful trends could not be extracted.

Competitive oxidative addition studies by ESI-MS spectroscopy

1) Competitive reactions in 1:1:1 ratio between **7**, **L2**, and **L3**

In a glovebox, 7.6 mg of **7** (0.012 mmol) was dissolved in 5 mL of 50:50 mixture of MeCN:THF. Separately, 1 equiv of ligands (**L2** or **L3**, 0.012 mmol) relative to **7** was dissolved in a 5 mL 50:50 mixture of MeCN:THF. 1 mL of each ligand stock, 1 mL of the solvent mixture, and tetrabutylammonium bromide as an internal standard (3.9 mg, 0.012 mmol) were transferred to the same 4 mL vial sealed with a screw cap with hole with PTFE/silicone septum. The mixture was fully mixed to afford homogeneous solution. Aliquot was taken to measure ESI-MS before adding solution **7**. 1 mL of the **7** solution was taken up in an airtight syringe, and the septum of the cuvette was punctured but not dispensed. The apparatus was removed from the glovebox and the solution of **7** was injected rapidly into the ligand solution. The mixture was shaken hard for 10 seconds then aliquot was taken to measure ESI-MS. We compared the relative amounts of ligands before and after the addition of **7**. In this study, 7.4% of the **L3** ligand and 9.8% of the **L2** ligand were used. The consumed ligand ratio (**L3**/**L2**; 7.4/9.8 ≈ 0.75) is similar to the initial rate ratio between (**L3**/**L2**; $1.35\times10^{-3}/1.76\times10^{-3} \approx 0.77$) obtained from the independent IR measurements. Also, Hammett parameter obtained from this competitive reaction ($\log(7.4/9.8)/\Delta\sigma_p = -0.13$) is similar to ρ (= -0.11) from the independent reaction. This indicates that the electronic properties of the ligands have a negligible influence on the overall reaction rate, as the competitive reaction results align closely with the Hammett analysis.

Table S3. Normalized peak intensity ratio (%) between ligands participated in the competitive reaction (**7**: **L3**: **L2** = 1: 1: 1).

	**L3**	**L2**
Before the reaction	100	100
After the reaction (10 seconds)	92.6	90.2
Consumed ligand	7.4	9.8

2) Competitive reactions in 0.25:1:1 ratio between **7**, **L2**, and **L3**

In a glovebox, 7.6 mg of **7** (0.012 mmol) was dissolved in 5 mL of 50:50 mixture of MeCN:THF. Separately, 1 equiv of ligands (**L2** or **L3**, 0.012 mmol) relative to **7** was dissolved in a 5 mL 50:50 mixture of MeCN:THF. 1 mL of each ligand stock, 0.75 mL of the solvent mixture, and tetrabutylammonium bromide as an internal standard (3.9 mg, 0.012 mmol) were transferred to the same 4 mL vial sealed with a screw cap with hole with PTFE/silicone septum. The mixture was fully mixed to afford homogeneous solution. Aliquot was taken to measure ESI-MS before adding solution **7**. 0.25 mL of the **7** solution was taken up in an airtight syringe, and the septum of the cuvette was punctured but not dispensed. The apparatus was removed from the glovebox and the solution of **7** was injected rapidly into the ligand solution. The mixture was shaken hard for 10 seconds then aliquot was taken to measure ESI-MS. We compared the relative amounts of ligands

before and after the addition of **7**. In this study, 3.6% of the **L3** ligand and 4.3% of the **L2** ligand were used. When it is compared with the competitive reaction of 1:1:1 ratio (**7**: **L3**: **L2**), half of each ligand was consumed in the same reaction time (for **L3**: 3.6/7.4 = 0.49; for **L2**: 4.3/9.8 = 0.44). It indicates that the reaction is half-order reaction of **7**.

Table S4. Normalized peak intensity ratio (%) between ligands participated in the competitive reaction (**7**: **L3**: **L2** = 0.25: 1: 1).

	**L3**	**L2**
Before the reaction	100	100
After the reaction (10 seconds)	96.4	95.7
Consumed ligand	3.6	4.3

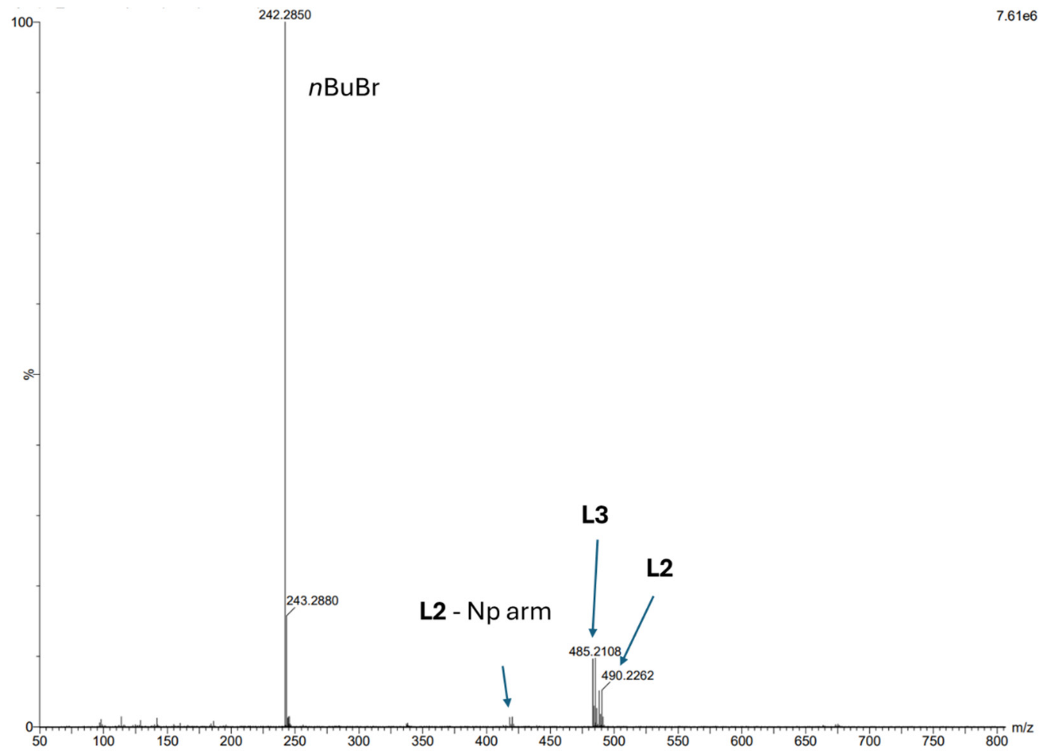

Figure S40. ESI-MS spectra for 1:1 ratio of **L2** and **L3** in a 50:50 mixture of MeCN:THF before adding **7** at room temperature (*n*BuBr as an internal standard).

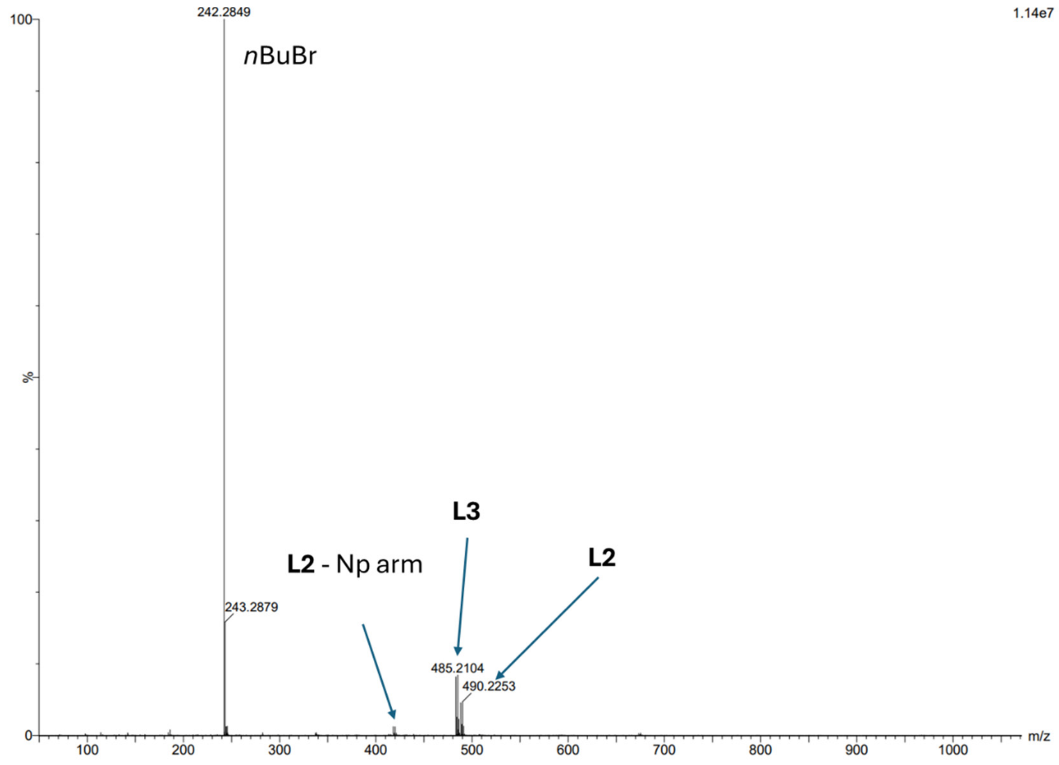

Figure S41. ESI-MS spectra for 10 seconds after adding **7** (1 equiv) to the ligand mixture.

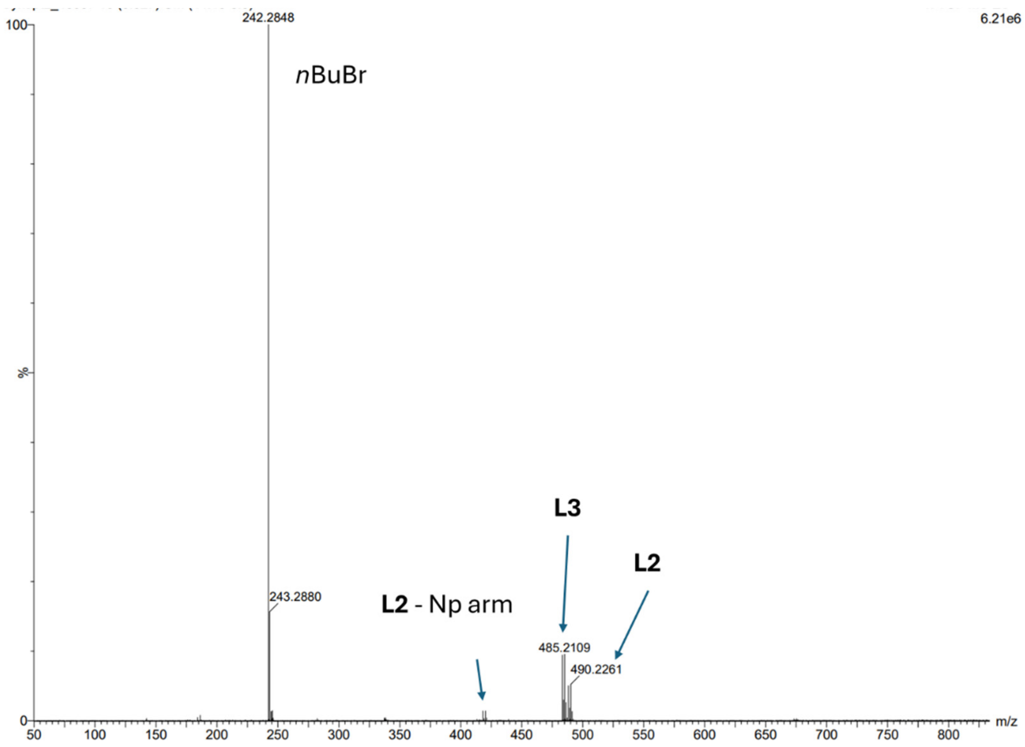

Figure S42. ESI-MS spectra for 1:1 ratio of **L2** and **L3** in a 50:50 mixture of MeCN:THF before adding **7** at room temperature (*n*BuBr as an internal standard).

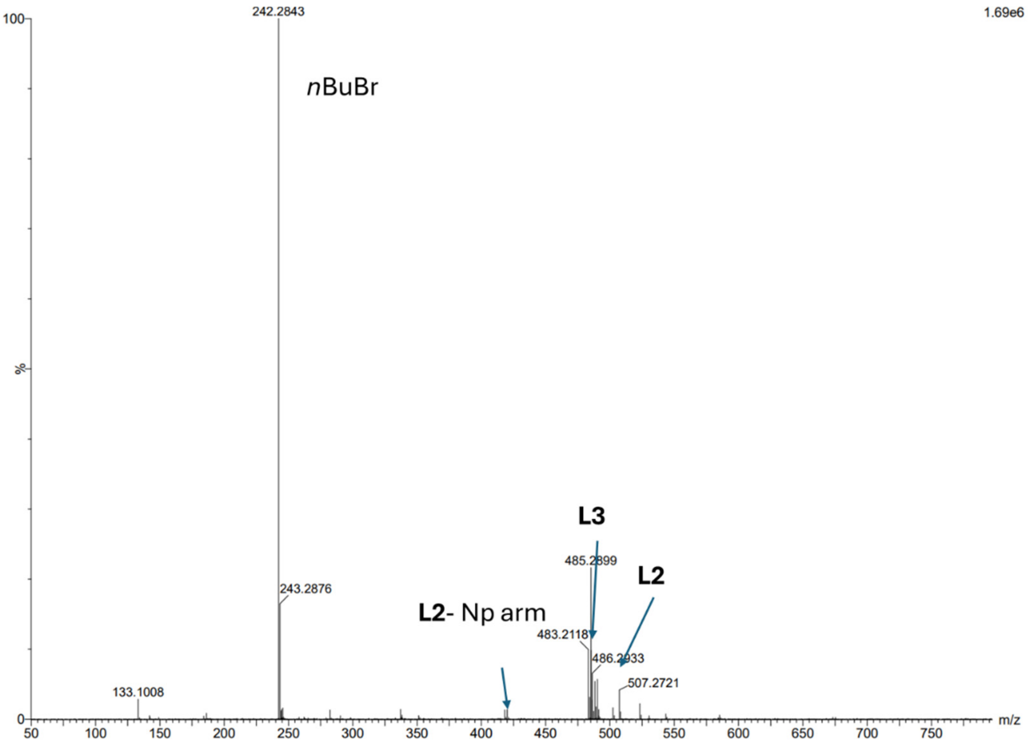

Figure S43. ESI-MS spectra for 10 seconds after adding **7** (0.25 equiv) to the ligand mixture.

V. Solid State Structure Determinations

General information

Suitable crystals were mounted on MiTeGen cryoloops in random orientations in a Bruker Kappa Apex-II CCD X-ray diffractometer equipped with an Oxford Cryostream LT device and a fine focus Mo Kα radiation X-ray source (λ = 0.71073 Å). Preliminary unit cell constants were determined with a set of 36 narrow frame scans. Typical data sets consist of combinations of ϖ and φ scan frames with a typical scan width of 0.5° and a counting time of 15−30 s/frame at a crystal-to-detector distance of 3.5 cm. The collected frames were integrated using an orientation matrix determined from the narrow frame scans. Apex II and SAINT software packages[8] used for data collection and data integration. Analysis of the integrated data did not show any decay. Final cell constants were determined by global refinement of xyz centroids of reflections from the complete data sets. Collected data were corrected for systematic errors using SADABS (Bruker Analytical X-Ray, Madison, WI, 2008) based on the Laue symmetry using equivalent reflections. Crystal data and intensity data collection parameters are listed in corresponding tables of each complex. Structure solutions and refinement were carried out using the SHELXTL-PLUS software package. [9] The structures were solved by direct methods and refined successfully. Full matrix least-squares refinements were carried out by minimizing $\Sigma w(Fo^2-Fc^2)^2$. The non-hydrogen atoms were refined anisotropically to convergence. The hydrogen atoms were treated using an appropriate riding model.

X-ray structure determination of [(L1)Pd^{III}Br(MeCN)]PF_6 (1)

Table S5. Crystal data and structure refinement for **1**.

Identification code	l1916
Empirical formula	$C_{27}H_{39}BrF_6N_4PPd$
Formula weight	750.90
Temperature/K	99.92
Crystal system	monoclinic
Space group	$P2_1/c$
a/Å	17.2224(11)
b/Å	14.0824(8)
c/Å	13.6044(8)
α/°	90
β/°	109.835(3)
γ/°	90
Volume/Å^3	3103.8(3)
Z	4
ρ_{calc}g/cm^3	1.607
μ/mm^{-1}	1.997
F(000)	1516.0
Crystal size/mm^3	0.296 × 0.212 × 0.194
Radiation	MoKα (λ = 0.71073)
2Θ range for data collection/°	3.832 to 72.734
Index ranges	$-23 \leq h \leq 27$, $-23 \leq k \leq 15$, $-21 \leq l \leq 20$
Reflections collected	78674
Independent reflections	13753 [R_{int} = 0.0408, R_{sigma} = 0.0412]
Data/restraints/parameters	13753/0/368
Goodness-of-fit on F^2	1.044
Final R indexes [I>=2σ (I)]	R_1 = 0.0356, wR_2 = 0.0841
Final R indexes [all data]	R_1 = 0.0543, wR_2 = 0.0908
Largest diff. peak/hole / e Å^{-3}	2.10/-0.69

Table S6. Bond Lengths for **1**.

Atom	Atom	Length/Å	Atom	Atom	Length/Å
Pd1	Br1	2.4753(2)	C6	C7	1.502(2)
Pd1	N1	2.0458(13)	C8	C9	1.516(2)
Pd1	N2	2.3431(13)	C9	C10	1.377(2)
Pd1	N3	2.3377(14)	C10	C11	1.393(3)
Pd1	N4	2.1935(15)	C11	C12	1.388(3)
Pd1	C1	1.9510(16)	C12	C13	1.389(2)
N1	C9	1.341(2)	C13	C14	1.519(2)
N1	C13	1.337(2)	C16	C17	1.553(2)
N2	C7	1.482(2)	C17	C18	1.536(3)
N2	C8	1.487(2)	C17	C19	1.530(2)
N2	C16	1.495(2)	C17	C20	1.539(3)
N3	C14	1.484(2)	C21	C22	1.556(2)
N3	C15	1.487(2)	C22	C23	1.538(3)
N3	C21	1.489(2)	C22	C24	1.537(3)
N4	C26	1.127(2)	C22	C25	1.524(3)
C1	C2	1.381(2)	C26	C27	1.460(3)
C1	C6	1.383(2)	P1	F1	1.5948(14)
C2	C3	1.393(2)	P1	F2	1.5960(16)
C2	C15	1.497(2)	P1	F3	1.5877(16)
C3	C4	1.393(2)	P1	F4	1.5977(15)
C4	C5	1.391(2)	P1	F5	1.5897(14)
C5	C6	1.394(2)	P1	F6	1.5919(15)

Table S7. Bond Angles for **1**.

Atom	Atom	Atom	Angle/°	Atom	Atom	Atom	Angle/°
N1	Pd1	Br1	173.45(4)	N2	C7	C6	111.35(13)
N1	Pd1	N2	79.58(5)	N2	C8	C9	115.45(13)
N1	Pd1	N3	80.15(5)	N1	C9	C8	117.68(14)
N1	Pd1	N4	88.25(5)	N1	C9	C10	120.06(15)
N2	Pd1	Br1	100.52(3)	C10	C9	C8	122.21(15)
N3	Pd1	Br1	97.79(4)	C9	C10	C11	118.61(16)
N3	Pd1	N2	153.96(5)	C12	C11	C10	120.27(16)
N4	Pd1	Br1	98.21(4)	C11	C12	C13	118.50(16)
N4	Pd1	N2	97.97(5)	N1	C13	C12	119.79(15)
N4	Pd1	N3	97.56(5)	N1	C13	C14	118.11(14)
C1	Pd1	Br1	91.34(5)	C12	C13	C14	122.06(15)
C1	Pd1	N1	82.20(6)	N3	C14	C13	115.73(13)
C1	Pd1	N2	80.55(6)	N3	C15	C2	111.54(13)
C1	Pd1	N3	80.67(6)	N2	C16	C17	121.89(13)
C1	Pd1	N4	170.44(6)	C18	C17	C16	112.81(14)
C9	N1	Pd1	118.83(11)	C18	C17	C20	107.93(15)
C13	N1	Pd1	118.18(11)	C19	C17	C16	113.61(14)
C13	N1	C9	122.65(14)	C19	C17	C18	109.05(15)
C7	N2	Pd1	102.59(9)	C19	C17	C20	108.78(15)
C7	N2	C8	113.39(13)	C20	C17	C16	104.36(14)
C7	N2	C16	113.30(12)	N3	C21	C22	121.37(14)
C8	N2	Pd1	104.22(9)	C23	C22	C21	104.31(14)
C8	N2	C16	113.73(13)	C24	C22	C21	112.47(14)
C16	N2	Pd1	108.41(9)	C24	C22	C23	107.06(16)
C14	N3	Pd1	104.09(10)	C25	C22	C21	113.79(14)
C14	N3	C15	113.74(13)	C25	C22	C23	108.81(15)
C14	N3	C21	113.63(13)	C25	C22	C24	109.98(16)
C15	N3	Pd1	102.01(9)	N4	C26	C27	179.01(19)
C15	N3	C21	113.00(13)	F1	P1	F2	90.19(8)
C21	N3	Pd1	109.19(9)	F1	P1	F4	89.57(7)
C26	N4	Pd1	163.41(14)	F2	P1	F4	179.75(9)
C2	C1	Pd1	117.58(12)	F3	P1	F1	179.37(10)
C2	C1	C6	123.81(15)	F3	P1	F2	89.97(9)
C6	C1	Pd1	117.77(12)	F3	P1	F4	90.27(9)
C1	C2	C3	117.66(14)	F3	P1	F5	89.45(10)
C1	C2	C15	117.30(14)	F3	P1	F6	91.92(10)
C3	C2	C15	124.90(14)	F5	P1	F1	89.94(9)
C2	C3	C4	120.15(15)	F5	P1	F2	89.98(8)
C5	C4	C3	120.59(16)	F5	P1	F4	90.07(8)

Atom	Atom	Atom	Angle/°	Atom	Atom	Atom	Angle/°
C4	C5	C6	120.08(16)	F5	P1	F6	178.56(10)
C1	C6	C5	117.68(14)	F6	P1	F1	88.68(8)
C1	C6	C7	117.16(14)	F6	P1	F2	89.60(10)
C5	C6	C7	124.94(15)	F6	P1	F4	90.34(9)

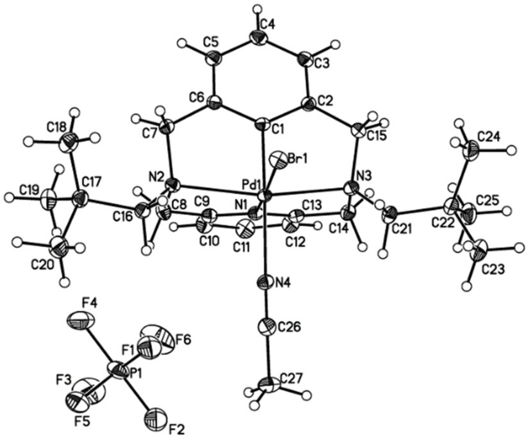

Figure S44. ORTEP representation of **1** shown at 50% probability.

X-ray structure determination of [(L4)Pd^{III}Br(MeCN)]PF_6 (4)

Table S8. Crystal data and structure refinement for **4**.

Identification code	l4015
Empirical formula	$C_{56}H_{79}N_{11}F_{12}P_2Br_2Pd_2$
Formula weight	1568.86
Temperature/K	99.95
Crystal system	monoclinic
Space group	$P2_1/c$
a/Å	8.9197(8)
b/Å	16.6937(14)
c/Å	21.5770(19)
α/°	90
β/°	94.891(5)
γ/°	90
Volume/Å^3	3201.2(5)
Z	2
ρ_{calc}g/cm^3	1.628
μ/mm^{-1}	1.941
F(000)	1584.0
Crystal size/mm^3	0.457 × 0.139 × 0.062
Radiation	MoKα (λ = 0.71073)
2Θ range for data collection/°	3.088 to 51.62
Index ranges	$-7 \leq h \leq 10$, $-20 \leq k \leq 20$, $-26 \leq l \leq 26$
Reflections collected	42335
Independent reflections	6025 [R_{int} = 0.0889, R_{sigma} = 0.0709]
Data/restraints/parameters	6025/18/402
Goodness-of-fit on F^2	1.027
Final R indexes [I>=2σ (I)]	R_1 = 0.0448, wR_2 = 0.0850
Final R indexes [all data]	R_1 = 0.0865, wR_2 = 0.1030
Largest diff. peak/hole / e Å^{-3}	1.12/-0.85

Table S9. Bond Lengths for **4**.

Atom	Atom	Length/Å	Atom	Atom	Length/Å
Pd1	Br1	2.4835(7)	C10	C11	1.372(7)
Pd1	N1	2.064(4)	C11	C12	1.394(7)
Pd1	N2	2.376(4)	C12	C13	1.387(7)
Pd1	N3	2.416(4)	C13	C14	1.500(7)
Pd1	N4	2.172(5)	C16	C17	1.525(7)
Pd1	C1	1.950(5)	C16	C18	1.533(7)
N1	C9	1.334(6)	C16	C19	1.527(7)
N1	C13	1.328(6)	C20	C21	1.526(7)
N2	C14	1.482(6)	C20	C22	1.529(7)
N2	C15	1.512(6)	C20	C23	1.527(7)
N2	C16	1.529(6)	C24	C25	1.458(8)
N3	C7	1.466(7)	P1	F1	1.585(3)
N3	C8	1.485(6)	P1	F2	1.588(3)
N3	C20	1.522(6)	P1	F3	1.599(3)
N4	C24	1.124(7)	P1	F4	1.603(3)
C1	C2	1.384(7)	P1	F5	1.598(3)
C1	C6	1.389(7)	P1	F6	1.588(3)
C2	C3	1.400(7)	C1S	C2S	1.41(2)
C2	C15	1.505(7)	C1S	N1S	1.180(19)
C3	C4	1.371(7)	C3S	C4S	1.43(2)
C4	C5	1.394(7)	C3S	N2S	1.17(2)
C5	C6	1.382(7)	C5S	C6S	1.42(2)
C6	C7	1.497(7)	C5S	N3S	1.18(2)
C8	C9	1.498(7)	C7S	C8S	1.43(3)
C9	C10	1.387(7)	C7S	N4S	1.17(2)

Table S10. Bond Angles for **4**.

Atom	Atom	Atom	Angle/°	Atom	Atom	Atom	Angle/°
N1	Pd1	Br1	175.22(12)	N3	C8	C9	116.0(4)
N1	Pd1	N2	75.47(15)	N1	C9	C8	117.8(4)
N1	Pd1	N3	78.24(15)	N1	C9	C10	119.3(5)
N1	Pd1	N4	92.52(16)	C10	C9	C8	122.5(4)
N2	Pd1	Br1	105.62(10)	C11	C10	C9	118.9(5)
N2	Pd1	N3	146.51(14)	C10	C11	C12	120.6(5)
N3	Pd1	Br1	99.01(10)	C13	C12	C11	117.9(5)
N4	Pd1	Br1	91.90(11)	N1	C13	C12	120.0(5)
N4	Pd1	N2	99.06(15)	N1	C13	C14	116.7(4)
N4	Pd1	N3	102.47(15)	C12	C13	C14	123.2(5)
C1	Pd1	Br1	89.48(15)	N2	C14	C13	111.3(4)
C1	Pd1	N1	86.10(18)	C2	C15	N2	112.6(4)
C1	Pd1	N2	80.63(18)	N2	C16	C18	110.5(4)
C1	Pd1	N3	77.22(18)	C17	C16	N2	109.0(4)
C1	Pd1	N4	178.62(19)	C17	C16	C18	109.9(4)
C9	N1	Pd1	119.4(3)	C17	C16	C19	108.8(4)
C13	N1	Pd1	117.5(3)	C19	C16	N2	109.0(4)
C13	N1	C9	123.1(4)	C19	C16	C18	109.7(4)
C14	N2	Pd1	99.4(3)	N3	C20	C21	110.1(4)
C14	N2	C15	108.9(4)	N3	C20	C22	110.7(4)
C14	N2	C16	111.7(4)	N3	C20	C23	108.6(4)
C15	N2	Pd1	104.4(3)	C21	C20	C22	109.5(4)
C15	N2	C16	111.7(4)	C21	C20	C23	109.0(4)
C16	N2	Pd1	119.6(3)	C23	C20	C22	108.9(4)
C7	N3	Pd1	97.6(3)	N4	C24	C25	178.6(6)
C7	N3	C8	109.3(4)	F1	P1	F2	90.11(18)
C7	N3	C20	114.1(4)	F1	P1	F3	178.9(2)
C8	N3	Pd1	102.7(3)	F1	P1	F4	90.17(18)
C8	N3	C20	111.6(4)	F1	P1	F5	89.5(2)
C20	N3	Pd1	120.1(3)	F1	P1	F6	90.89(19)
C24	N4	Pd1	171.3(5)	F2	P1	F3	90.71(19)
C2	C1	Pd1	119.1(4)	F2	P1	F4	179.6(2)
C2	C1	C6	123.8(5)	F2	P1	F5	90.41(19)
C6	C1	Pd1	117.0(4)	F2	P1	F6	89.79(18)
C1	C2	C3	116.9(5)	F3	P1	F4	89.02(18)
C1	C2	C15	120.0(5)	F5	P1	F3	89.86(19)
C3	C2	C15	122.8(5)	F5	P1	F4	89.3(2)
C4	C3	C2	120.4(5)	F6	P1	F3	89.79(19)
C3	C4	C5	121.0(5)	F6	P1	F4	90.50(19)

Atom	Atom	Atom	Angle/°	Atom	Atom	Atom	Angle/°
C6	C5	C4	120.1(5)	F6	P1	F5	179.6(2)
C1	C6	C7	117.1(5)	N1S	C1S	C2S	173(2)
C5	C6	C1	117.5(5)	N2S	C3S	C4S	175(2)
C5	C6	C7	125.2(5)	N3S	C5S	C6S	174(3)
N3	C7	C6	110.6(4)	N4S	C7S	C8S	162(6)

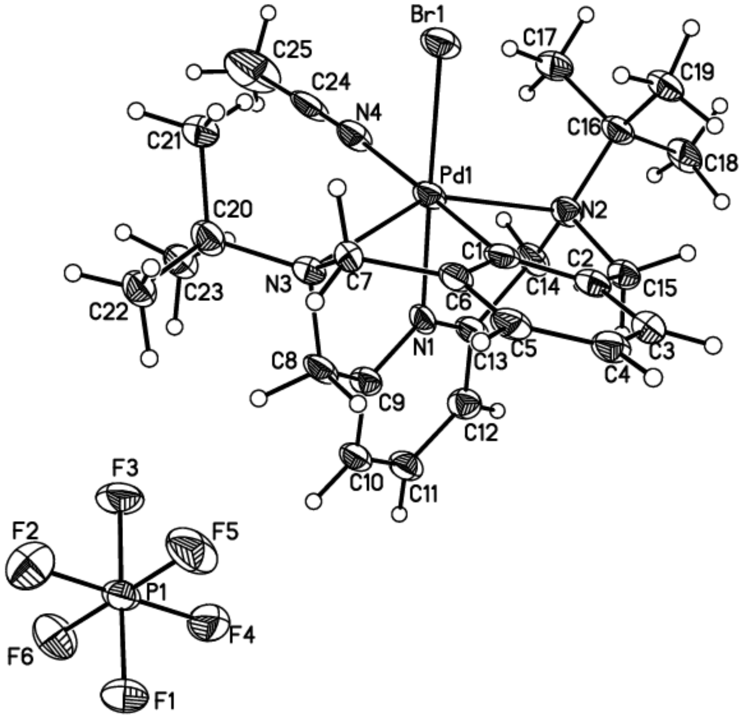

Figure S45. ORTEP representation of **4** shown at 50% probability. Solvent molecules are omitted for clarity.

VI. Computational Details

Geometry optimizations and frequency calculations were performed at the M06 level of theory utilizing the Stuttgart/Dresden ECP (SDD) basis set using the Gaussian 16 package.[10-12] No constraints were forced on the geometry optimization. Following the experimental procedure, acetonitrile: THF solvent mixture (1:1 v/v) was used as the solvent (ε=21.5569), and solvation was modeled utilizing the SMD variant of the IEFPCM SCRF model.[13]

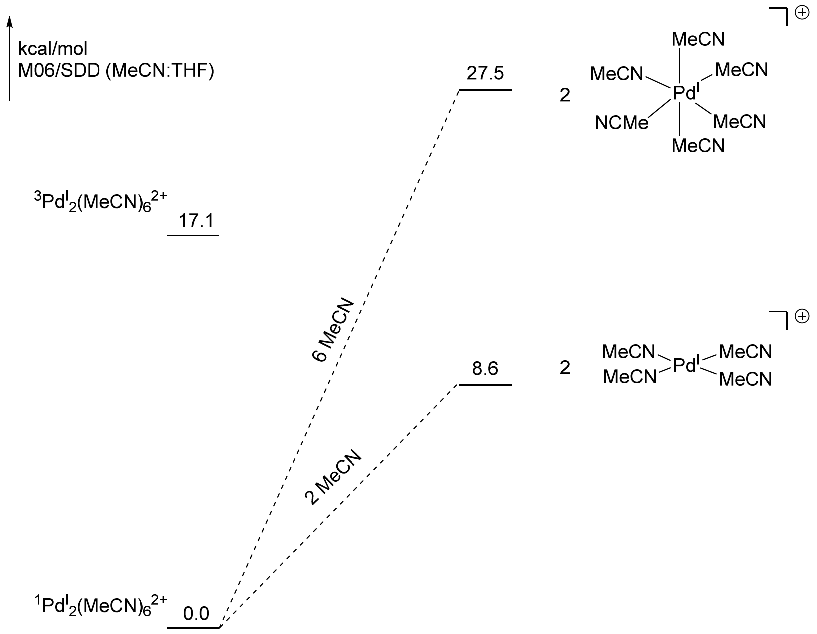

Figure S46. Calculated energy profiles for the conversion of $Pd^{I}_2(MeCN)_6^{2+}$ (**7**) to 2 equiv of $Pd^{I}(MeCN)_x^+$ (x= 4 or 6).

Cartesian coordinates and energies (Hartrees) of optimized complexes obtained by DFT at the M06/SDD level of theory:

MeCN

Energy: -132.6198154

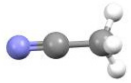

Charge: 0; Multiplicity: 1
N 5.567443884 12.6890842032 14.2598541121
C 5.3921648212 13.1831113496 13.2046002853
C 5.1748171225 13.7956171839 11.8962939569
H 6.0113163058 13.5735082453 11.2283506154
H 5.0864225241 14.8808810597 11.9940835929
H 4.2568353425 13.4111979582 11.4438174375

$[Pd^{I}_{2}MeCN_{6}]^{2+}$ (complex **7**)

Energy: -1051.317256

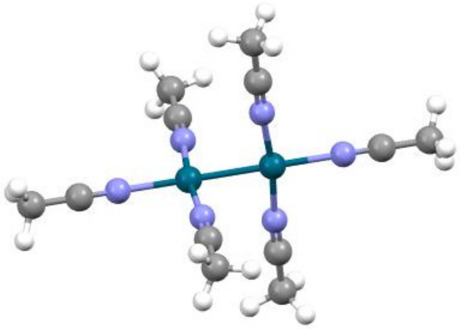

Charge: 2; Multiplicity: 1
Pd 0.3733129646 2.3103351234 3.009754277
Pd 1.6187790429 0.8924708074 1.3141512102
N -0.6801097997 3.5086384445 4.4427683983
N 2.6722557163 -0.3058785901 -0.1188878999
N 1.7894043049 3.6962392217 2.7066172209
N -0.9217567314 0.7868096943 3.1485556324
N 0.462308108 1.7579935191 -0.0754775624
N 2.6545561008 0.1640159197 2.8678970706
C -1.2544883956 4.1596958346 5.2227301535
C -1.9700429767 4.96931747 6.1941972837
C 3.2463145244 -0.9584006302 -0.8978624686
C 3.9613000111 -1.7714395272 -1.8668942196
C 2.5842488672 4.5382149595 2.5718225362
C 3.5715263247 5.5897578277 2.407605978
C -1.706990406 -0.0660229127 3.2708607592
C -2.6878754694 -1.124745799 3.4271068845
C -0.1811154604 2.2274949033 -0.9266216723

C -0.979689737 2.8089560827 -1.9903877837
C 3.28879634 -0.2952976794 3.7314102465
C 4.0814800675 -0.8703587855 4.8030360897
H -1.8420493312 4.5611795398 7.2004662011
H -1.5951485919 5.9963199408 6.187433308
H -3.0382589225 4.9904498587 5.9619645736
H 3.6904476138 -2.8249601046 -1.7560906398
H 3.7172069061 -1.4570546783 -2.8851710051
H 5.0411091437 -1.6743801093 -1.725234119
H 4.0435970345 5.8229268173 3.366096157
H 4.3493953157 5.2810840347 1.7040341755
H 3.0999618669 6.4995720438 2.025796113
H -3.6880463207 -0.7574901781 3.1807668807
H -2.4553150526 -1.9655639046 2.7680958353
H -2.6983488502 -1.4857160596 4.4593407676
H -1.6868859665 3.5383015721 -1.5863341215
H -0.3373704514 3.314495035 -2.7168728996
H -1.544008004 2.0311719433 -2.5124476384
H 3.9302452635 -1.9523660842 4.8510990871
H 5.1450738142 -0.6785760162 4.635962035
H 3.7983801365 -0.4375885344 5.7663141556

$[Pd^{I}MeCN_4]^+$

Energy: -658.2715784

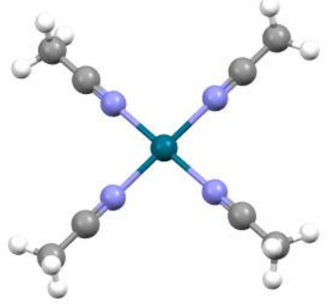

Charge: 1; Multiplicity: 2
Pd 1.6108441416 0.9534578439 1.4411777619
N 2.7877059092 -0.1509256847 -0.0165732233
N 0.6207008831 2.0284041094 -0.169653973
N 2.6019834152 -0.1208094923 3.0518156297
C 3.418961772 -0.7457749919 -0.7985975892
C 4.2053421164 -1.4871974409 -1.7717867861
C 0.086837677 2.6069395499 -1.0323128024
C -0.5791687284 3.3273946617 -2.1058833856
C 3.1368066697 -0.6991042515 3.9140392199
C 3.8033738434 -1.4201376477 4.9868808645
H 4.0452576901 -2.5620413578 -1.6525665924
H 3.9194429648 -1.2056813165 -2.7886223812
H 5.2703308904 -1.2776972099 -1.6412148117
H -0.638869438 4.3933456017 -1.8707862376
H -0.0292223421 3.2106647372 -3.0433810151
H -1.5935983871 2.9458798707 -2.2493112955

H 3.4830330081 -2.4652914022 5.0006372407
H 4.8876366024 -1.3906711285 4.8504525342
H 3.5645476488 -0.9719245817 5.9548701095
N 0.4321246744 2.0561577307 2.8988192999
C -0.2016680668 2.6487632557 3.6804925564
C -0.9915048242 3.3872902127 4.6530821581
H -0.7344587415 3.0769743941 5.6692334549
H -0.8039754419 4.4603819377 4.5612064739
H -2.0579779367 3.2059426002 4.4953347896

$[Pd^{I}MeCN_6]^+$

Energy: -923.4962827

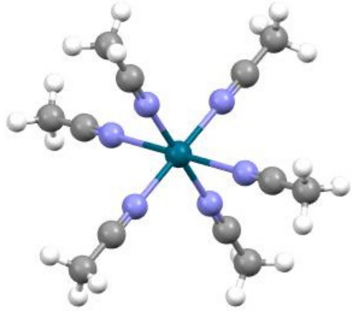

Charge: 1; Multiplicity: 2
Pd 5.6694810618 3.2739246331 5.7855587377
N 4.768415545 4.8125329948 7.0589253483
C 4.289275445 5.6324788441 7.738329138
C 3.6925548093 6.6547713646 8.5851265323
N 4.4869439982 1.2060458069 7.456844107
C 4.014993724 0.3745975838 8.1384597182
C 3.4286405895 -0.6584156492 8.9859024475
N 7.4590975758 3.2759093814 7.0505636363
C 8.4112475039 3.2789954135 7.72649527
C 9.5975893148 3.2839101573 8.5696051741
N 6.5703905331 1.7350111927 4.5123007627
C 7.0492223979 0.9148181639 3.8329766291
C 7.6456174663 -0.1077666905 2.9863031968
N 6.8532967228 5.3412579394 4.116380519
C 7.3265481993 6.1723670072 3.4352626515
C 7.914367868 7.2049505167 2.5883196095
N 3.8798176111 3.2716050427 4.5206901578
C 2.9276071641 3.2682501695 3.8448431575
C 1.7411799617 3.2631667599 3.0018551488
H 3.9922998143 6.5117555845 9.6267343759
H 2.6016508591 6.6078896962 8.53083521
H 4.0123839734 7.650505678 8.2665252114
H 3.4189004406 -0.3370049185 10.0307777587
H 4.004755011 -1.5848430523 8.9151919686
H 2.4002082655 -0.8673472916 8.6795817683
H 9.3248734807 3.1165442196 9.6150121426
H 10.1154475306 4.2439704683 8.4960947002
H 10.2886464076 2.4943316556 8.2627021488

H 7.3442851958 0.033989363 1.9449857133
H 8.7365557374 -0.0599366687 3.0390380078
H 7.3270739164 -1.10346703 3.3063141562
H 7.9244461935 6.8836724614 1.5434099555
H 7.3390238779 8.1318848098 2.65878006
H 8.9427873697 7.4129423866 2.8953062236
H 2.0137282949 3.4308979914 1.9564633916
H 1.2236751254 2.30289669 3.0751532471
H 1.0498710147 4.0524073248 3.3090520186

$[Pd^{I}_2MeCN_6]^{2+}$

Energy: -1051.289991

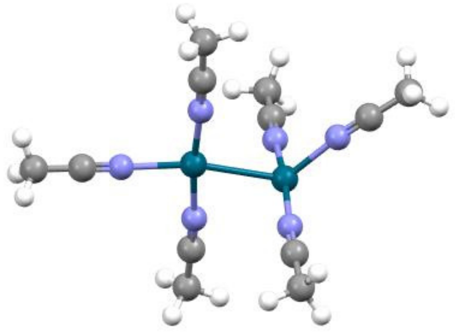

Charge: 2; Multiplicity: 3
Pd 0.7093259594 2.07230974 3.7513091164
Pd 1.5331308906 0.9785490372 1.2504685104
N -0.8456116779 3.5611384043 3.8702000822
N 2.4951973936 -0.1369352226 -0.3547576285
N 2.2468319254 3.465304789 3.6335898707
N -0.5906042535 0.4708774899 4.0020760361
N 0.2837241525 1.9970897202 -0.023776913
N 2.6926652044 0.0692551395 2.7172860835
C -1.6686406296 4.3660216827 4.0652138993
C -2.6905864559 5.3674024784 4.3230841533
C 2.9981576275 -0.7244301298 -1.2303878739
C 3.6230275604 -1.4555339647 -2.3209969344
C 3.0962160101 4.2653027211 3.6158958786
C 4.1486666349 5.265367337 3.6007450967
C -1.3321664929 -0.4097821812 4.192540069
C -2.2587971377 -1.5005659898 4.4366420817
C -0.3961857596 2.5398850112 -0.8013775121
C -1.2336238437 3.2051873581 -1.7841300217
C 3.3906792199 -0.495477861 3.4656613628
C 4.2663491862 -1.20733977 4.3796006475
H -2.7675022595 5.5602720745 5.3967411302
H -2.4440537848 6.3065193369 3.8206289135
H -3.6643968817 5.0244485201 3.9635346903
H 3.2584138578 -2.485908871 -2.3485497729
H 3.3938777234 -0.9817035594 -3.2791976146
H 4.7090122253 -1.4746672455 -2.1963702355
H 4.5393395787 5.422159277 4.6100631529
H 4.9718403981 4.947507545 2.9552361936

H 3.7641518731 6.2185523265 3.2276092096
H -3.2793500097 -1.1974397012 4.1872188329
H -1.9974117155 -2.3705871811 3.8282982187
H -2.2335383468 -1.7930119096 5.4900901987
H -1.955251577 3.86464262 -1.2949298836
H -0.6210214841 3.8050405824 -2.4628679069
H -1.7842277423 2.4685795873 -2.3758022861
H 4.090016864 -2.2846618053 4.3144984832
H 5.3137713489 -1.0141093218 4.1315304211
H 4.0907734179 -0.8896570646 5.4109812504

VII. References

1. Zhou, W.; Schultz, J. W.; Rath, N. P.; Mirica, L. M., Aromatic Methoxylation and Hydroxylation by Organometallic High-Valent Nickel Complexes. *J. Am. Chem. Soc.* **2015,** *137* (24), 7604-7607.
2. Zhou, W.; Watson, M. B.; Zheng, S.; Rath, N. P.; Mirica, L. M., Ligand effects on the properties of Ni(III) complexes: aerobically-induced aromatic cyanation at room temperature. *Dalton Trans.* **2016,** *137*, 15886-15893.
3. Zhou, W.; Zheng, S. A.; Schultz, J. W.; Rath, N. P.; Mirica, L. M., Aromatic Cyanoalkylation through Double C-H Activation Mediated by Ni(III). *J. Am. Chem. Soc.* **2016,** *138* (18), 5777-5780.
4. Evans, D. F., The determination of the paramagnetic susceptibility of substances in solution by nuclear magnetic resonance. *J. Chem. Soc.* **1959**, 2003-5.
5. De Buysser, K.; Herman, G. G.; Bruneel, E.; Hoste, S.; Van Driessche, I., Determination of the Number of Unpaired Electrons in Metal-Complexes. A Comparison Between the Evans' Method and Susceptometer Results. *Chem. Phys.* **2005,** *315* (3), 286-292.
6. Bain, G. A.; Berry, J. F., Diamagnetic Corrections and Pascal's Constants. *J. Chem Ed.* **2008,** *85* (4), 532-536.
7. Murahashi, T.; Nagai, T.; Okuno, T.; Matsutani, T.; Kurosawa, H., Synthesis and ligand substitution reactions of a homoleptic acetonitrile dipalladium(I) complex. *Chem. Comm.* **2000,** (17), 1689-1690.
8. Bruker. APEXIII. Bruker AXS, Inc. *Madison, Wisconsin, USA* **2018**.
9. Sheldrick, G. M., Bruker-SHELXTL. *Acta Cryst. A* **2008,** *64*, 112-122.
10. Frisch, M. J. T., G. W.; Schlegel, H. B.; Scuseria, G. E.; Robb, M. A.; Cheeseman, J. R.; Scalmani, G.; Barone, V.; Petersson, G. A.; Nakatsuji, H.; Li, X.; Caricato, M.; Marenich, A. V.; Bloino, J.; Janesko, B. G.; Gomperts, R.; Mennucci, B.; Hratchian, H. P.; Ortiz, J. V.; Izmaylov, A. F.; Sonnenberg, J. L.; Williams-Young, D.; Ding, F.; Lipparini, F.; Egidi, F.; Goings, J.; Peng, B.; Petrone, A.; Henderson, T.; Ranasinghe, D.; Zakrzewski, V. G.; Gao, J.; Rega, N.; Zheng, G.; Liang, W.; Hada, M.; Ehara, M.; Toyota, K.; Fukuda, R.; Hasegawa, J.; Ishida, M.; Nakajima, T.; Honda, Y.; Kitao, O.; Nakai, H.; Vreven, T.; Throssell, K.; Montgomery, J. A., Jr.; Peralta, J. E.; Ogliaro, F.; Bearpark, M. J.; Heyd, J. J.; Brothers, E. N.; Kudin, K. N.; Staroverov, V. N.; Keith, T. A.; Kobayashi, R.; Normand, J.; Raghavachari, K.; Rendell, A. P.; Burant, J. C.; Iyengar, S. S.; Tomasi, J.; Cossi, M.; Millam, J. M.; Klene, M.; Adamo, C.; Cammi, R.; Ochterski, J. W.; Martin, R. L.; Morokuma, K.; Farkas, O.; Foresman, J. B.; Fox, D. J., Gaussian 16, Revision C.01. *Gaussian, Inc., Wallingford CT* **2016**.
11. Zhao, Y.; Truhlar, D. G., The M06 suite of density functionals for main group thermochemistry, thermochemical kinetics, noncovalent interactions, excited states, and transition elements: two new functionals and systematic testing of four M06-class functionals and 12 other functionals. *Theor. Chem. Acc.* **2008,** *120* (1-3), 215-241.
12. Andrae, D.; Haussermann, U.; Dolg, M.; Stoll, H.; Preuss, H., Energy-Adjusted Abinitio Pseudopotentials for the 2nd and 3rd Row Transition-Elements. *Theor. Chim. Acta* **1990,** *77* (2), 123-141.
13. Marenich, A. V.; Cramer, C. J.; Truhlar, D. G., Universal Solvation Model Based on Solute Electron Density and on a Continuum Model of the Solvent Defined by the Bulk Dielectric Constant and Atomic Surface Tensions. *J. Phys. Chem. B* **2009,** *113* (18), 6378-6396.
